# Infectious particle identity determines dissemination and disease outcome for the inhaled human fungal pathogen *Cryptococcus*

**DOI:** 10.1371/journal.ppat.1007777

**Published:** 2019-06-27

**Authors:** Naomi M. Walsh, Michael R. Botts, Andrew J. McDermott, Sébastien C. Ortiz, Marcel Wüthrich, Bruce Klein, Christina M. Hull

**Affiliations:** 1 Department of Biomolecular Chemistry, School of Medicine and Public Health, University of Wisconsin-Madison, Madison, Wisconsin, United States of America; 2 Department of Medical Microbiology and Immunology, School of Medicine and Public Health, University of Wisconsin-Madison, Madison, Wisconsin, United States of America; 3 Department of Pediatrics, School of Medicine and Public Health, University of Wisconsin-Madison, Madison, Wisconsin, United States of America; Rutgers New Jersey Medical School, UNITED STATES

## Abstract

The majority of invasive human fungal pathogens gain access to their human hosts via the inhalation of spores from the environment into the lung, but relatively little is known about this infectious process. Among human fungal pathogens the most frequent cause of inhaled fatal fungal disease is *Cryptococcus*, which can disseminate from the lungs to other tissues, including the brain, where it causes meningoencephalitis. To determine the mechanisms by which distinct infectious particles of *Cryptococcus* cause disseminated disease, we evaluated two developmental cell types (spores and yeast) in mouse models of infection. We discovered that while both yeast and spores from several strains cause fatal disease, there was a consistently higher fungal burden in the brains of spore-infected mice. To determine the basis for this difference, we compared the pathogenesis of avirulent yeast strains with their spore progeny derived from sexual crosses. Strikingly, we discovered that spores produced by avirulent yeast caused uniformly fatal disease in the murine inhalation model of infection. We determined that this difference in outcome is associated with the preferential dissemination of spores to the lymph system. Specifically, mice infected with spores harbored *Cryptococcus* in their lung draining lymph nodes as early as one day after infection, whereas mice infected with yeast did not. Furthermore, phagocyte depletion experiments revealed this dissemination to the lymph nodes to be dependent on CD11c+ phagocytes, indicating a critical role for host immune cells in preferential spore trafficking. Taken together, these data support a model in which spores capitalize on phagocytosis by immune cells to escape the lung and gain access to other tissues, such as the central nervous system, to cause fatal disease. These previously unrealized insights into early interactions between pathogenic fungal spores and lung phagocytes provide new opportunities for understanding cryptococcosis and other spore-mediated fungal diseases.

## Author summary

Little is known about how inhaled spores from human fungal pathogens cause infections and spread to other parts of the body. The most frequent cause of inhaled fatal fungal disease is *Cryptococcus*, which causes meningitis. To understand how *Cryptococcus* causes disease, we evaluated pathogenesis of two types of cells (spores and yeast) in a mouse model of infection. We compared yeast strains that cannot cause disease to the spore offspring they produced during sexual reproduction. We discovered that parental yeast that are not virulent produced spores that were fully virulent and caused fatal meningitis. This difference was associated with movement of spores to the lymph system; mice infected with spores had *Cryptococcus* in their lung draining lymph nodes, but mice infected with yeast did not. Furthermore, when we infected mice that lacked immune cells in their lungs, no spores were found in their lymph nodes. This indicates that instead of protecting mice from the spore infection, the immune cells moved spores out of the lung to the lymph system where spores could then spread to the brain. These insights into interactions between pathogenic fungal spores and lung immune cells provide new opportunities for understanding cryptococcosis and other spore-mediated fungal diseases.

## Introduction

Through the act of breathing, the mammalian lung is regularly exposed to a wide variety of airborne particles, such as dust, air pollutants, and microbes. Both physical and immunological barriers have evolved to keep the lung clear of foreign agents and facilitate efficient respiration. Inert particles such as dust and pollen are cleared effectively, as are most microbes. However, many disease-causing organisms such as bacteria and fungi that gain entry via the lung have developed strategies for evading clearance, allowing them to colonize the lung and ultimately escape and disseminate to other tissues [1].

One particularly successful inhaled human pathogen is *Cryptococcus*. This environmental fungus is found in association with soil, tree bark, and bird droppings, and upon inhalation can escape the lung and cause fatal fungal meningoencephalitis [2]. Immunocompromised patients are most at risk of developing cryptococcosis, but disease among healthy individuals is on the rise worldwide [3]. Mortality rates range from 20–80%, depending on patient status and antifungal drug availability [3–5]. Hundreds of thousands of people a year develop cryptococcosis and die, due in large part to limited treatment options for invasive fungal diseases and challenges in treating brain infections [6].

Much attention has been paid to the propensity of this fungus to invade the central nervous system and the mechanisms by which it crosses the blood brain barrier during disseminated cryptococcosis; however, the mechanisms by which *Cryptococcus* escapes the lung to cause disseminated disease remain poorly understood [7,8]. Proposed infectious particles in human cryptococcal disease are yeast (its vegetative growth form) and spores (the products of sexual development) [9–11]. Because of the technical challenges associated with isolating large populations of pure spores, the vast majority of studies of *Cryptococcus* pathogenesis have been carried out with the more tractable yeast form.

Yeast were used to develop the mouse models of cryptococcal infection and disease over the last fifty years, and yeast have been shown to harbor unusual virulence traits [12]. Surprisingly, yeast are not phagocytosed efficiently by phagocytes in the absence of opsonization, which appears to be largely due to the presence of a polysaccharide coat. This covering (a.k.a. capsule) on the cell surface effectively masks immunoreactive epitopes, preventing efficient phagocytosis by host immune cells [13]. Once phagocytosed, however, *Cryptococcus* yeast can reside and grow within acidified phagolysosomes, presumably avoiding additional immune surveillance [14]. Finally, yeast can escape phagocytes by causing phagocyte rupture or through non-lytic exit via exocytosis [15]. Each of these traits (phagocytosis resistance, intracellular survival, and efficient escape) has been associated with the ability of *Cryptococcus* to cause disease.

More recently, spores were also shown to cause disease in mice in a mouse intranasal model of infection [9,10]. In contrast to yeast, however, spores are phagocytosed rapidly and efficiently in vitro in the absence of opsonization [10,16]. The spore surface harbors exposed beta-1,3-glucan, mannose, chitin, and other immunoreactive carbohydrates that presumably facilitate recognition and uptake by phagocytes [17]. Once inside phagocytes, spores can germinate into yeast and grow vegetatively. These spore-derived yeast replicate, reside inside phagolysosomes, and escape phagocytes in a manner indistinguishable from phagocytosed yeast [10].

Given the distinct interactions that spores and yeast display with immune phagocytes in vitro, we hypothesized that spores and yeast would interact with mammalian lung immune phagocytes differently in vivo, leading to differences in disease progression and/or outcome.

Initial experiments did not appear to support this hypothesis because intranasal infections of mice with either spores or yeast from highly virulent strains of *Cryptococcus* resulted in nearly identical survival curves [10]. While these experiments showed definitively that spores can be disease-causing particles, they did not inform mechanisms of pathogenesis. To determine whether spores and yeast cause disease via similar or distinct mechanisms, we carried out a series of mouse infections with spores and yeast derived from several different backgrounds of *Cryptococcus*. We monitored fungal dissemination kinetics, tissue distributions and burdens, interactions with phagocytes, host cytokine production, and host mortality.

We discovered that, in fact, disease progression and outcome are profoundly affected by the nature of the infectious particles (spore vs. yeast), and the degree of this effect is more pronounced in strains with virulence profiles that resemble natural isolates. In all strains tested, intranasal infection with *Cryptococcus* spores led to higher fungal burdens in the brain (but not other tissues) at endpoint relative to yeast. In strains from backgrounds in which the yeast are avirulent in the intranasal model of infection, mice did not develop disease when infected with yeast (as expected); however, the spores produced by those avirulent yeast strains caused 100% fatal disease in mice. Furthermore, in all cases, spore-infected mice showed much earlier and higher rates of dissemination from the lung via the lung draining lymph node, and this dissemination was dependent on lung phagocytes. These data indicate that spore interactions with host phagocytes specifically facilitate spore escape from the lung and promote dissemination to other tissues.

## Results

### Spores result in higher fungal burdens in the brain than yeast in a mouse intranasal model of infection

While spores have long been presumed infectious particles of *Cryptococcus*, it was only relatively recently confirmed that spores are virulent in a mouse model of infection. Spores produced by the congenic *Cryptococcus neoformans* var. *grubii* (a.k.a. *C*. *neoformans*, serotype A) strains KN99**a** and KN99α, cause fatal disease with kinetics indistinguishable from those of their yeast parents [9,10]. To determine whether spores of other *Cryptococcus* strains were also capable of causing disease, we produced spores from crosses between the virulent type strain H99 (α) and the Botswanan clinical isolate BT63 (**a**) (both serotype A) [18] and tested both yeast and spores in mice. Mice were infected intranasally with 1x10^5^ **a** yeast (BT63), 1x10^5^ α yeast (H99), 1x10^5^ total **a** + α yeast (a 1:1 mixture of BT63 and H99) or 1x10^5^ spores (from a BT63 x H99 cross) with the expectation that each haploid spore germinates into one haploid yeast [17]. Survival times and fungal burdens in the brain and lung at endpoint were assessed.

**Fig 1:**
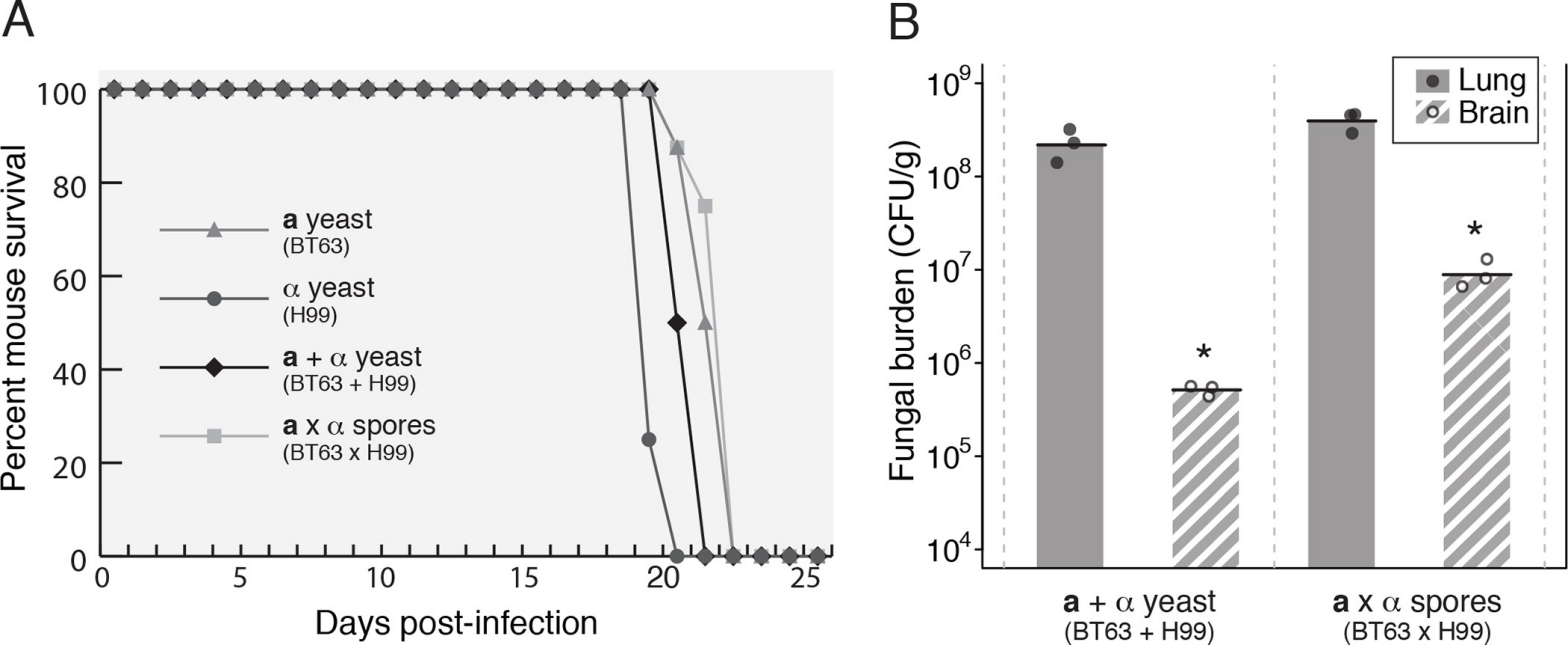
Spores result in higher fungal burdens in the brain than yeast in a mouse intranasal model of infection. (A) Representative survival plot of mice (8 per group) infected intranasally with 1x10^5^ BT63 (**a**) yeast alone (triangles), 1x10^5^ H99 (α) yeast alone (circles), 1x10^5^ yeast as a 1:1 mixture of both strains (BT63 + H99; diamonds), or 1x10^5^ spores derived from a cross between strains (BT63 x H99; squares). (B) Fungal burdens (as colony-forming units per gram of tissue) at endpoint in the lungs (gray bars) and brains (striped bars) of mice infected with 1x10^5^ yeast in a 1:1 mixture of both parent strains (BT63 + H99) or with 1x10^5^ spores derived from a cross between strains (BT63 x H99) (3 mice per group). Each circle represents an individual mouse. *p = 0.011 for brain comparisons; for lung and all other tissues, p ≥ 0.10 (using a Student’s t-test). Data are representative of multiple biological replicates (see Materials and Methods).

As was seen with KN99**a** and KN99α previously (10), the BT63 and H99 strains caused disease as both yeast and spores. We observed no statistical difference in the mean time to death for spore-infected (21 days) or yeast-infected (20 days) mice (Fig 1A). Also consistent with our previous observations for KN99**a** and KN99α, the BT63 x H99 spores yielded higher brain burdens at end point than yeast. Spore-infected mice harbored ~18-fold higher CFUs in the brain than yeast-infected mice at 28 days post-infection (9.2x10^6^ vs. 5.2x10^5^ CFU/g, respectively, p = 0.011). There were no significant differences in numbers of CFUs from any other tissues (Fig 1B). Thus, these data demonstrate that infection with BT63 x H99 spores causes fatal disease with the same kinetics as infection with their parental yeast. These data also indicate that infection with spores is associated with higher fungal colonization of the brain at end point.

Given that spores isolated from crosses between the congenic KN99**a** and KN99α strains and the non-congenic H99 and BT63 strains behaved similarly with respect to virulence potential and brain CFUs, we concluded that 1) these phenotypes were not specific to the KN99**a**/α background and are likely relevant across *Cryptococcus* strains, and 2) the differences in brain fungal burdens between spore- and yeast-infected mice may reflect differences in disease progression between the two infectious cell types.

### Spores produced by avirulent yeast strains cause uniformly fatal cryptococcal meningoencephalitis in a mouse intranasal model of infection

**Fig 2:**
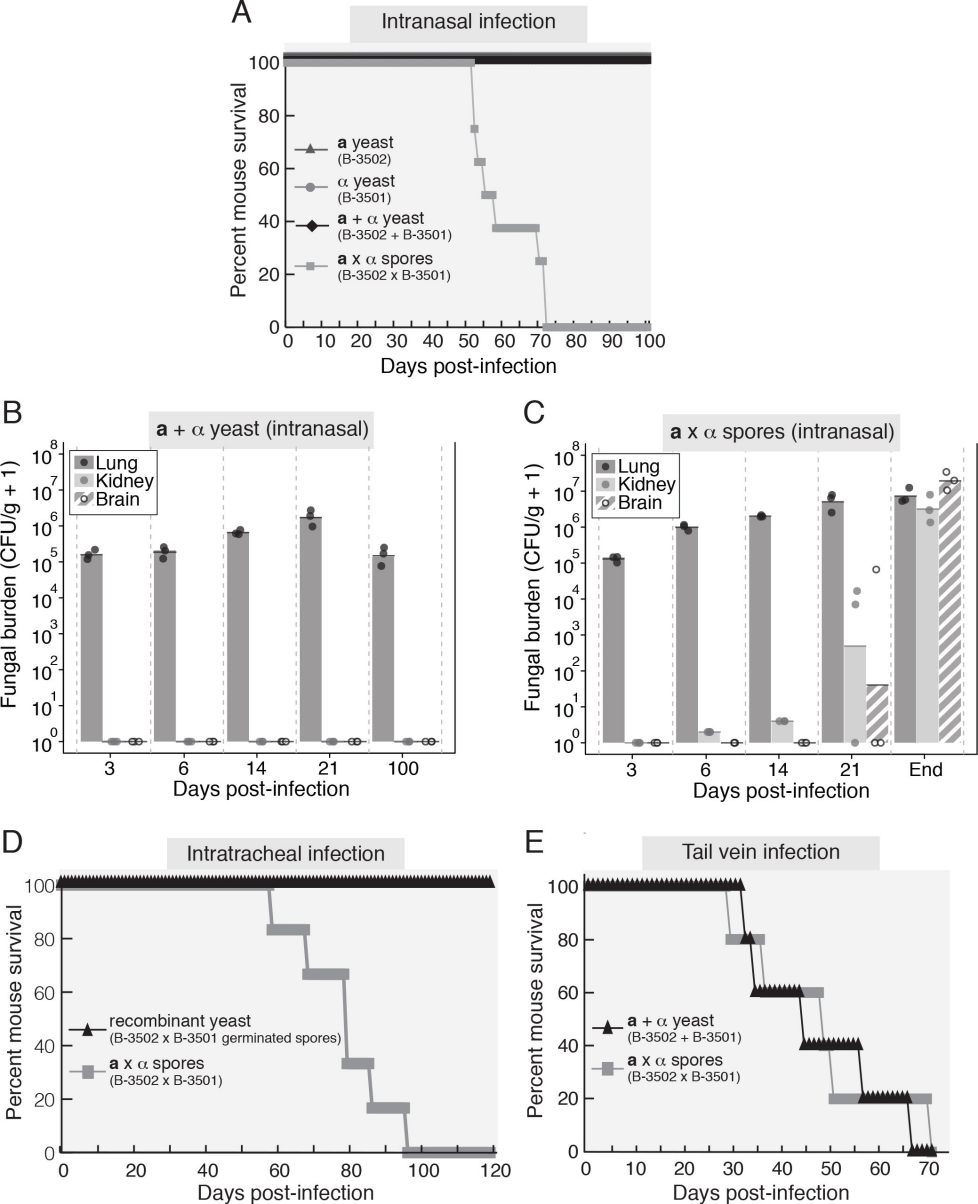
Spores produced by avirulent yeast strains cause uniformly fatal cryptococcal meningoencephalitis in a mouse intranasal model of infection. (A) Survival plot of mice (8 mice per group) infected intranasally with 2.5x10^5^ B-3502 yeast (triangles) or 2.5x10^5^ B-3501 yeast (circles), 2.5x10^5^ yeast as a 1:1 mixture of B-3502 and B-3501 (B-3502 + B-3501; diamonds), or 2.5x10^5^ spores derived from a cross between B-3502 and B-3501 (B-3502 x B-3501; squares). (B) Fungal burdens (as colony-forming units per gram of tissue plus 1) in the lung (dark gray bars), kidney (light gray bars), and brain (striped bars) at 3, 6, 14, 21, and 100 days after intranasal infection of mice with 2.5x10^5^ yeast as a 1:1 mixture of B-3502 and B-3501. Circles represent individual mice (3 mice per time point). (C) Fungal burdens (as colony-forming units per gram of tissue plus 1) in the lung (dark gray bars), kidney (light gray bars) and brain (striped bars) at 3, 6, 14, 21 days and at endpoint after intranasal infection of mice with 2.5x10^5^ spores derived from a cross between B-3502 and B-3501. Circles represent individual mice (3 mice per time-point). (D) Survival curves of 6 mice infected by intratracheal instillation with 1x10^5^ spores derived from a B-3502 x B-3501 cross (squares) or 5 mice infected with 1x10^5^ recombinant yeast (generated by germination of spores derived from a B-3502 x B-3501 cross into yeast; triangles). (E) Survival plots of mice infected by tail-vein injection with 1x10^6^ yeast in a 1:1 mixture (B-3502 + B-3501; triangles) or 1x10^6^ spores derived from a B-3502 x B-3501 cross (squares) (5 mice per group). Data in A, B, and C are representative of independent biological replicates (see Materials and Methods), and data in D and E are derived from single experiments.

In our experiments with the KN99**a**/KN99α and BT63/H99 strain pairs, the primary sign of disease in both spore- and yeast-infected mice was respiratory distress, at which point they were euthanized. Because infections with spores yielded higher brain fungal burdens than yeast at the time of euthanasia, we considered that the early respiratory disease produced by H99-derived strains prevented observation of the full effects of spore-mediated disease in the brain. We hypothesized that strains that do not cause rapid and fatal pulmonary disease would extend the timeline of disease progression and more clearly reveal differences between spore- and yeast-mediated disease. To test this hypothesis, we carried out spore- and yeast-mediated infections with strains known to show limited pulmonary virulence. Mice were infected with spores or yeast of *Cryptococcus neoformans* var. *neoformans* (a.k.a *C*. *deneoformans*) strains B-3502 (**a**) and B-3501 (α). Mice infected with 2.5x10^5^ yeast (**a** alone, α alone, or **a** + α) showed no signs of disease at any point during the experiment and were euthanized after 100 days (Fig 2A). Mice infected with 2.5x10^5^ spores, however, began to show signs of CNS disease starting at day 52, with all mice exhibiting evidence of CNS disease leading to euthanasia by day 75 (Fig 2A). This difference in disease outcome between yeast and spores indicates that by using avirulent yeast strains with limited effects on the lung, we were able to discern the natural progression of spore-mediated disease. In this case spores caused fatal meningoencephalitis when their yeast parents could not (p = 5.6x10^-5^ by a Log-Rank test for Kaplan-Meier survival analysis).

To determine potential differences in dissemination during yeast- and spore-mediated infections, we evaluated fungal organ burdens over the course of disease. We determined that yeast-infected mice harbored no fungal burden in any tissues outside the initial site of infection in the lungs at any time point (Fig 2B). While the lung burden did show an increase during the course of the experiment, by 100 days post-infection the number of fungi had dropped to levels similar to those observed very early after infection (Fig 2B). Histological analysis revealed analogous results in the lungs, showing an increased fungal burden and inflammation through day 21 and lower levels of inflammation and decreased numbers of cryptococcal cells at day 100. No abnormal pathology or fungal burden was observed in the brains of yeast-infected mice at any point (S1A Fig). These data demonstrate that there was no detectable dissemination of yeast from the lung to any other tissue tested and that respiratory infection with yeast was contained in the lung.

In contrast, mice infected with spores showed dissemination of *Cryptococcus* throughout all tissues evaluated (Fig 2C). As early as 14 days post-infection, *Cryptococcus* was detected in the kidneys of all mice, and by 21 days post-infection there was a detectable fungal burden in the brain of one animal. When spore-infected mice became moribund (between 50 and 75 days post-infection), the fungal burdens within the brain exceeded 10^7^ CFU/g in all animals (Fig 2C). It should be noted that spores are produced during sexual development in the environment (and not in mammalian hosts) and that spores are unable to replicate without first germinating into yeast (which can then replicate via budding). Thus, the observed fungal burdens are derived solely from spores that were introduced intranasally, germinated in the host, and grew vegetatively as yeast. Histological cross-sections of the lungs of spore-infected mice were very similar to those of yeast-infected mice through day 21. However, at endpoint (when spore-infected mice became moribund and were sacrificed), we observed increased inflammation and visible fungal burdens in the lungs as well as high numbers of fungal cells in the brain (S1B Fig). Taken together, these data indicate that infection with spores led to increased dissemination of *Cryptococcus* from the lungs to other tissues, relative to infection with yeast, leading to fatal meningoencephalitis.

### Differences in virulence between spores and yeast are due to infectious cell type-specific events that occur in the host lung

Because the spores for the spore vs. yeast infection experiments were derived from genetically distinct (non-congenic) yeast parents, we considered that even though the parental yeast were avirulent, it was formally possible that the difference in virulence between yeast and spores was a consequence of diverse genotypic combinations created during sexual development that could confer pathogenic abilities not harbored by either parent. To test the possibility that the spore population was more virulent than yeast due to the presence of virulent recombinant spore progeny in numbers sufficient to cause fatal disease, we germinated spores from B-3502 x B-3501 crosses into yeast and used those yeast to infect mice. Mice were infected with either 1x10^5^ spores purified from a B-3502 x B-3501 cross or 1x10^5^yeast derived by germinating spores from a B-3502 x B-3501 cross and monitored for signs of CNS disease. As was observed previously, spores from a B-3502 x B-3501 cross caused fatal disease in all mice. In contrast, yeast from germinated spores did not cause discernable disease in mice, supporting the hypothesis that the difference between spore- and yeast-mediated disease was dependent on the infectious cell type, rather than the presence of new virulent genotypes in the recombinant spore population (Fig 2D).

At the same time, we tested whether the difference between spore- and yeast-mediated disease was a consequence of more efficient spore entry into the brain via the nares by infecting mice using an intratracheal route of infection. As was seen with intranasal infections, mice infected with spores succumbed to fatal disease, whereas yeast-infected mice did not (Fig 2D). These data rule out the possibility that spore-specific properties (such as small size relative to yeast) led to enhanced disease via direct access to the brain through the nares. To confirm that spores and yeast reach the lung equally well after intranasal infection, we also infected mice intranasally with spores or yeast and assessed fungal burdens in the lungs 30 minutes after infection and found no difference. This result indicates that yeast and spores were equally capable of gaining initial entry to the lung (S2 Fig).

To test whether the differences between spore- and yeast-mediated disease were dependent on a lung route of infection, we introduced the yeast or spores directly into the bloodstream, bypassing the lung. Mice were inoculated via the tail vein with 1x10^6^ yeast or 1x10^6^ spores and monitored for signs of CNS disease. In contrast to the intranasally- and intratracheally-infected mice, all of the tail vein-infected mice succumbed to cryptococcosis by day 70 post-infection, regardless of whether they were infected with spores or yeast (Fig 2E). Fungal burden analysis revealed a high fungal burden in all tissues, including the brain, which did not differ significantly between yeast- or spore-infected mice. These data show that yeast and spores are equally capable of causing fatal disseminated disease when introduced directly into the bloodstream, indicating that spore- and yeast-mediated differences in disease are dependent on a lung route of infection.

Overall, we conclude from these experiments that spores and yeast are equally capable of infecting the murine lung, neither cell type accesses the brain directly from the nares, and both are fully capable of causing fulminant disease when introduced directly into the bloodstream. Thus, the differences in disease outcome resulting in higher brain burdens in spore-mediated infections are the results of events that occur in the lung prior to dissemination to the bloodstream.

### Spores and yeast do not elicit differences in lung immune polarization

Having established that the differences in disease between spores and yeast were dependent on both cell type and route of infection, we considered that the host response to spores could contribute to lung-dependent differences in disease. Previous studies have demonstrated that the Th1/Th2 bias of the pulmonary immune response is an important factor that determines cryptococcal disease progression and dissemination out of the lung [19]. To determine whether infection with spores or yeast drives altered polarization of the pulmonary immune response, cytokine production by T-cells and inflammatory infiltrate was evaluated following infection with spores or yeast in the murine lung. Mice were infected intranasally with yeast (B-3502 + B-3501) or spores (B-3502 x B-3501). Mice were also infected with H99 yeast for comparison to a known, highly virulent strain. At 11 days post infection, lungs were processed and evaluated to determine the total number of cells, the number of immune cells in the population, and the levels of immune cytokines following T-cell stimulation.

**Fig 3:**
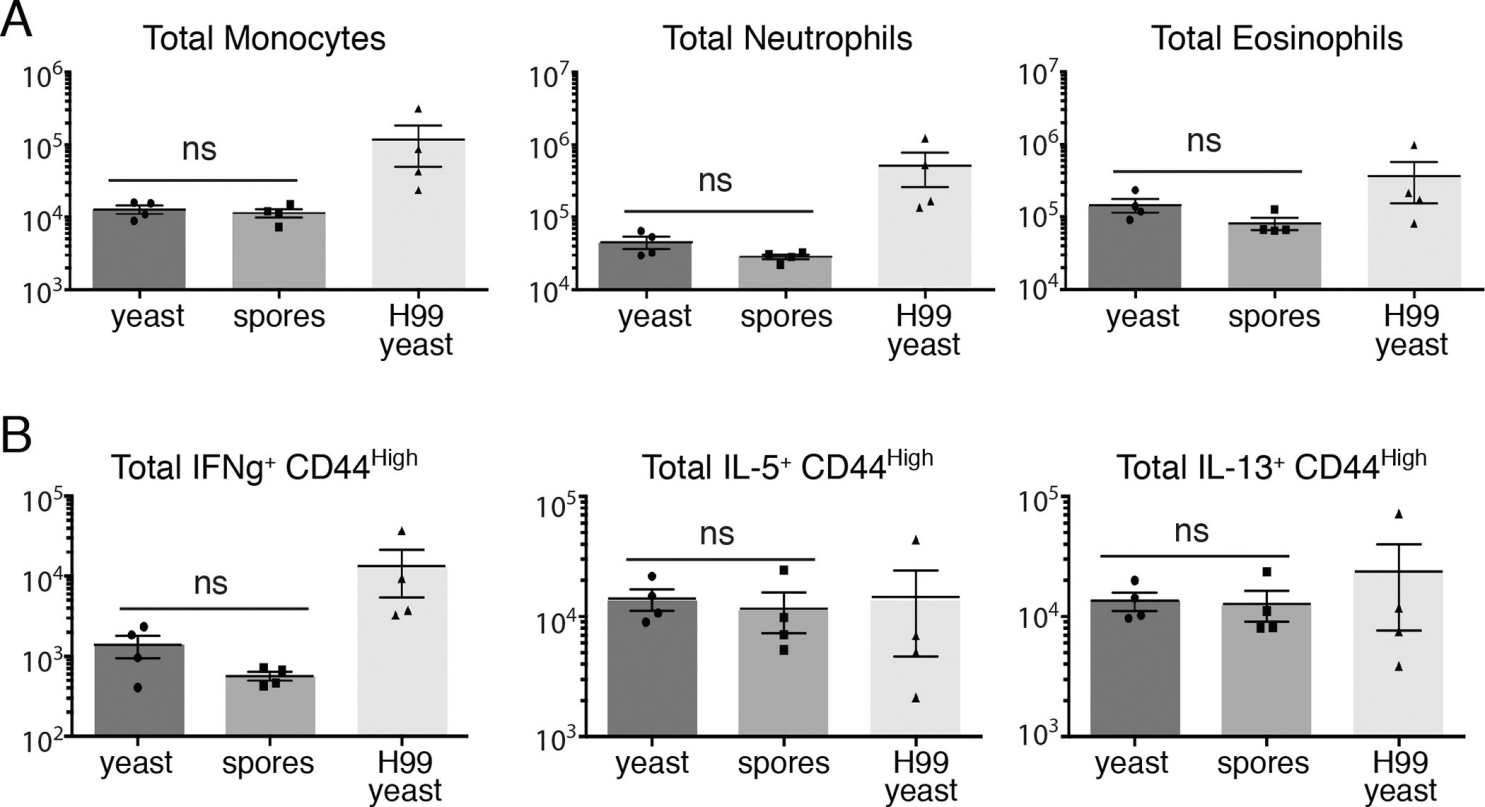
Yeast and spores of the same genetic background do not elicit differences in overall lung immune response. (A) Quantification of lung myeloid cell (monocytes, neutrophils, or eosinophils) recruitment elicited by intranasal infection with 2.5x10^5^ yeast of a 1:1 mixture of B-3502 + B-3501 (dark gray), 2.5x10^5^ spores from a B-3502 x B-3501 cross (gray), or 1x10^5^ H99 yeast (light gray) at 11 days post infection (4 mice per group). (B) Quantification of lung CD44+ T-cell cytokine production (interferon gamma (IFNg), interleukin-5 (IL-5), and interleukin-13 (IL-13)) following intranasal infection with *Cryptococcus* yeast or spores as described in (A). In all panels, the Y-axis indicates total number of host cells, error bars represent the mean ± standard error of the mean (SEM). ns = not significantly different (p > 0.1 in a Student's t-test). Circles, squares, and triangles represent individual mice. Data are derived from a single experiment.

As anticipated, mice infected with H99 yeast showed high numbers of total host cells in the lung, consistent with increased inflammation (S3 Fig). In contrast, B-3502 + B-3501 yeast showed relatively low numbers of host cells in the lung, indicating little inflammation, and this was also the case for mice infected with spores (B-3502 x B-3501) (S3 Fig). Staining of surface markers allowed us to identify various leukocytes (CD45+) by flow cytometry, including monocytes (SiglecF^low^, Ly6G^low^,CD11b^hi^,Ly6c^hi^), neutrophils (SiglecF^low^,CD11b^hi^,Ly6G^hi^), and eosinophils (SSC^hi^,SiglecF^hi^_,_MHCII^low^,CD11c^low^,CD11b^hi^). Again, as expected, mice infected with H99 yeast showed high numbers of all identified cell types, consistent with a robust immune response. In contrast, B-3502 + B-3501 yeast elicited much less immune cell recruitment. This was also the case for B-3502 x B-3501 spores, and the total numbers of monocytes, neutrophils, and eosinophils in spore- and yeast-infected mice were not different (Fig 3A).

To assess the balance of lung Th1/Th2 responses, we determined the cytokine response of CD44+ effector T-cells using ex vivo stimulation and flow cytometric analysis. Our analysis focused on identifying IFNgamma production as hallmark of Th-1 responses and improved outcomes in cryptococcal infection [20], and IL-13 as a marker of a non-protective Th2 response [21]. Higher expression of IL-5 has been shown to lead to increased pulmonary eosinophilia and higher cryptococcal burdens in the lungs of mice [22]. Overall, there were no significant differences in the cytokine responses assessed among the three groups of mice (Fig 3B). While mice infected with H99 yeast did show an apparent higher average number of CD44+IFNgamma+ cells, which are typically associated with a more protective TH1 response, we hypothesize that this is due to H99 yeast eliciting a higher immune response overall, relative to yeast and spores of B-3502 and B-3501.

From these data we conclude that spores and yeast (of the B-3502 and B-3501 backgrounds) do not initiate detectably different immune responses in the lung with both cell types eliciting similar immune cell recruitment and Th1/Th2 bias. While there could be more subtle differences in the immune responses to yeast- and spore-mediated infections that were not detected here, we conclude that the overall immune response mounted in the lungs is unlikely to be the deciding factor in the different disease outcomes observed between spore- and yeast-infected mice.

### *Cryptococcus* disseminates to the lung draining lymph node very early in spore-mediated infections

Having observed that overall lung immune response signaling in spore- and yeast-infected mice was largely the same, we hypothesized that direct interactions between cryptococcal cells and host cells would lead to differing dissemination patterns of spores and yeast. Previous studies showed that spores are more readily phagocytosed than yeast by alveolar macrophages in vitro (by ~10-fold) [10,23,24]. We further determined that preferential phagocytosis of spores occurs with a number of diverse phagocytic cell types, including RAW 264.7 cells (macrophages), JAWS II cells (dendritic-like cells), and murine bone marrow-derived phagocytes (S4 Fig). Based on these findings, we hypothesized that the difference in dissemination (and ultimately disease outcome after 50 days post-infection), between yeast and spores is dependent on their interactions with host lung phagocytes (that occur in the first day after infection). Respiratory immune cells are known to traffic to the lung draining lymph nodes within 24 hours following infection [25]; thus, we anticipated that the first point of dissemination of spores would be to the mediastinal lymph nodes. To test this hypothesis, we assessed dissemination of *Cryptococcus* in spore- and yeast-infected mice at early time points after infection. Mice were infected intranasally with spores or yeast, and fungal burdens in the lungs, lymph node, spleen, and blood were determined at 1, 5, and 7 days post infection.

**Fig 4:**
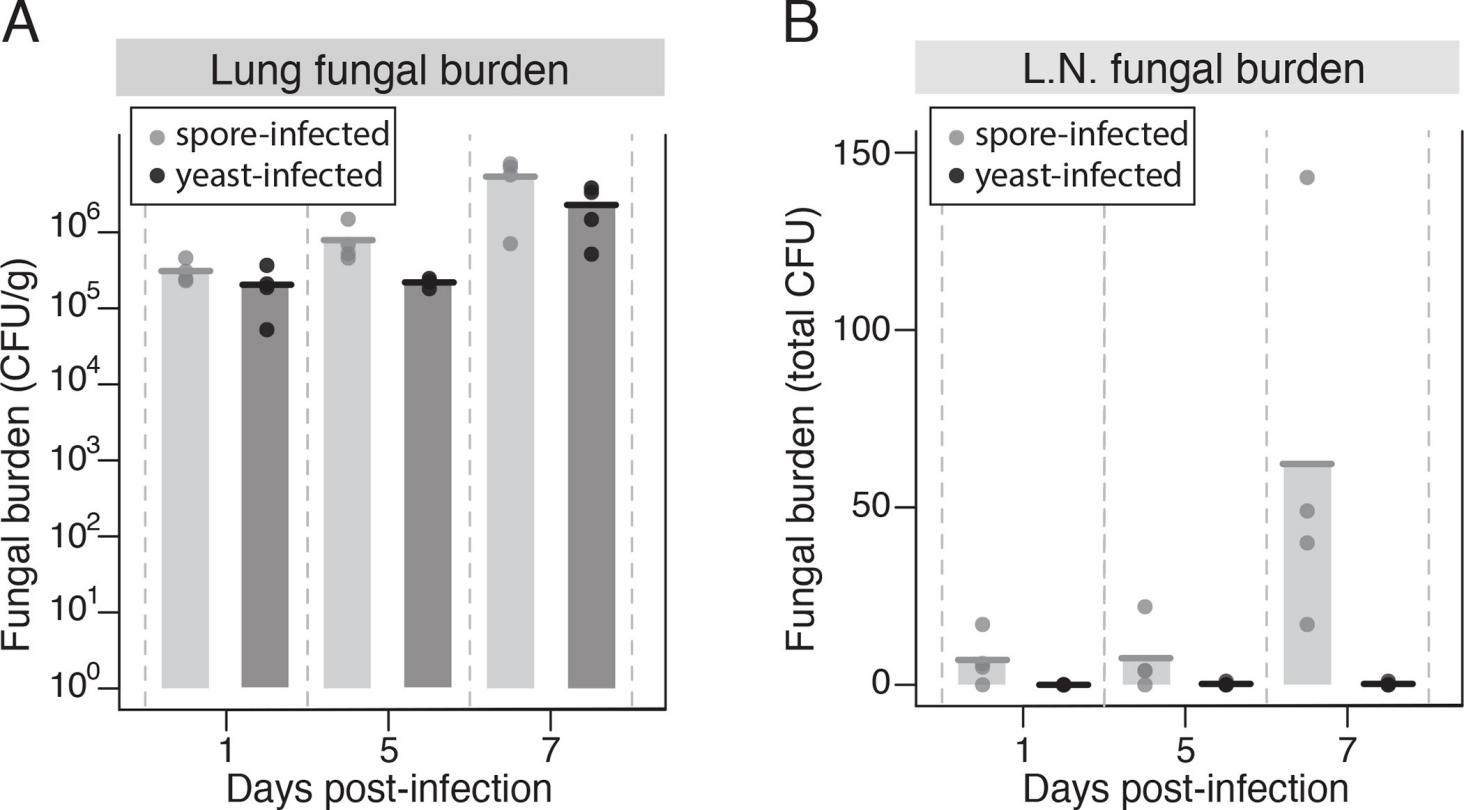
*Cryptococcus* disseminates to the draining lymph node very early in spore-mediated infections. (A) Fungal burden (colony forming units per gram of tissue) in the lungs of mice (3 per group) infected intranasally with 1x10^6^ yeast in a 1:1 mixture of JEC20 +J EC21 (dark gray) or 1x10^6^ spores derived from a JEC20 x JEC21 cross (light gray) after 1, 5, and 7 days of infection. (B) Fungal burden (total colony forming units per organ) in the lymph nodes of mice infected intranasally with 1x10^6^ yeast in a 1:1 mixture of JEC20 + JEC21 (dark gray) or 1x10^6^ spores derived from a JEC20 x JEC21 cross (light gray) after 1, 5, and 7 days of infection. Data are representative of multiple biological replicates (see S6 Fig).

Mice infected with spores or yeast showed similar levels of fungal burden in the lungs through day 7 post-infection and almost no dissemination to the spleen or blood at this time point (Fig 4A and S5 Fig). In contrast, however, there was a striking difference in dissemination between yeast and spores to the mediastinal lymph nodes. Mice that had been infected with yeast exhibited no fungal burden in their lymph nodes through day seven, whereas mice infected with spores showed an increasing burden in this tissue through the time course of the experiment (Fig 4B). As early as one-day post-infection there was a small, but markedly higher fungal burden in spore-infected mice than yeast-infected mice that was observed repeatedly and consistently across multiple experiments with various strains (S6 Fig). From these data, we conclude that 1) spores disseminate to the lung draining lymph node very early (less than 24 hours) post-infection before gaining access to any other tissues, and 2) spores disseminate to the lung draining lymph node much more readily than yeast. These findings establish a correlation between efficiency of phagocytosis in vitro and efficiency of dissemination to the lung draining lymph node, supporting the hypothesis that lung phagocytes engulf spores in vivo and traffic them out of the lung.

### Spore phagocytosis is dependent on germination state and intrinsic spore properties

Both spore- and yeast-mediated disease are caused by overwhelming fungal burdens composed of yeast, indicating that spores must germinate in vivo. In vitro, spore germination is a relatively rapid, synchronous process, occurring within 10–12 hours in the presence of rich media under laboratory conditions [26]. If germination occurs in a similar time frame in vivo, then one would predict that any differences in interactions of phagocytes with yeast and spores that influence dissemination would occur within the first day post-infection. To test this prediction and its effects on dissemination, we carried out experiments both in vitro and in vivo to determine the effects of germination on phagocytosis.

To assess spore germination in vivo, cells were recovered via bronchoalveolar lavage 18 hours after infection with spores or yeast, and host cells were lysed. Germination state of the recovered cryptococcal cells was evaluated by measuring their aspect ratios (ARs), where the AR of dormant, ovoid spores is approximately 0.6, and the AR of round yeast is close to 1.0 [26]. We found that cryptococcal cells recovered from spore- or yeast-infected mice were morphologically indistinguishable from one another (average AR = 0.94 and 0.96, respectively) and nearly identical to the in vitro yeast control (average AR = 0.95) (all p-values > 0.4) (S7 Fig). Furthermore, the germination states of these three groups (spore-infected, yeast-infected, and yeast control), were all significantly different morphologically than the in vitro ungerminated spore control (all p-values < 10^−10^) (S7 Fig). Thus, we conclude that the vast majority of spores introduced into the mouse lung germinate into yeast within 18 hours.

**Fig 5:**
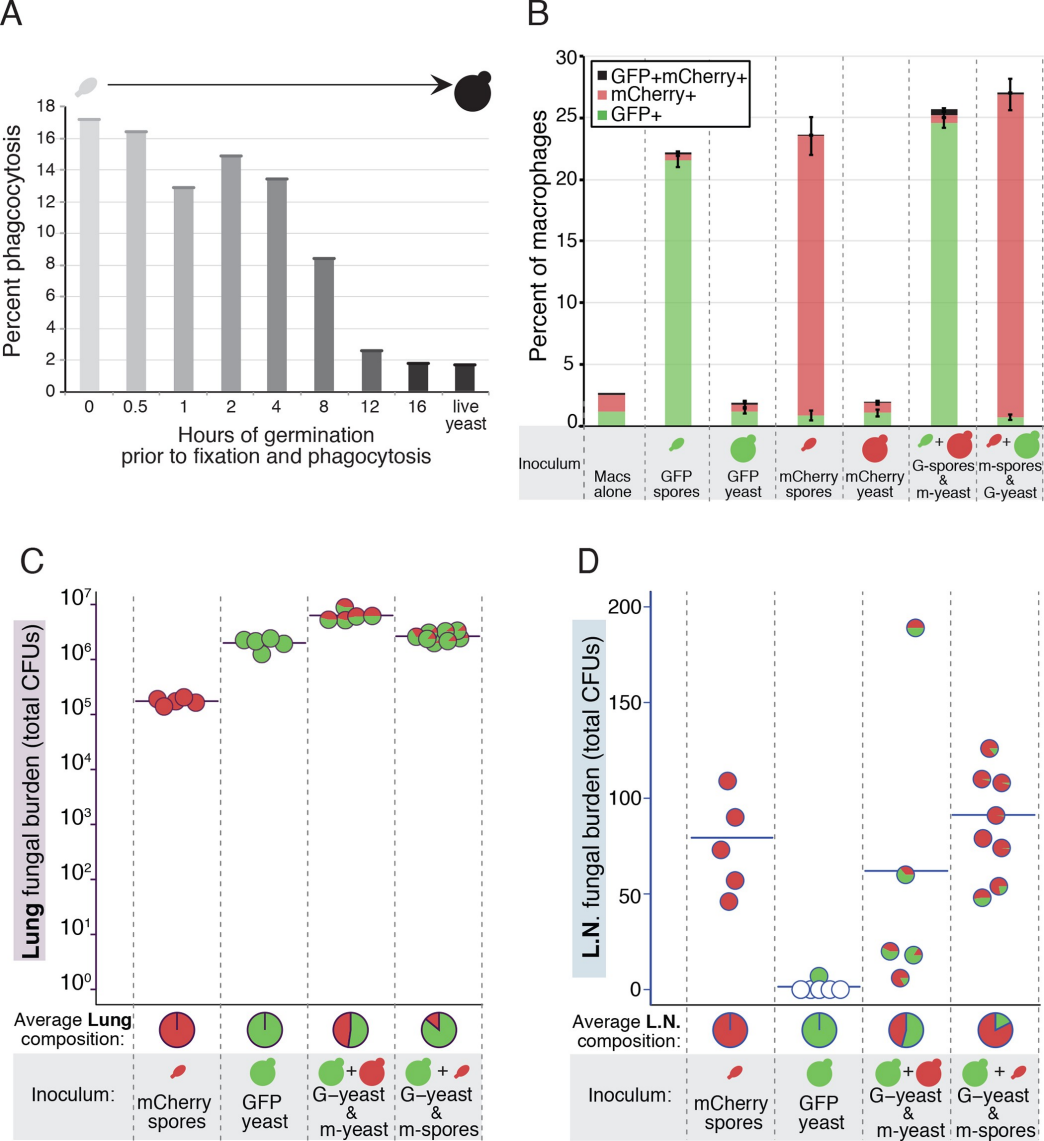
Spore phagocytosis and lymph node dissemination are dependent on intrinsic spore properties. (A) Phagocytosis of formaldehyde-fixed spores by RAW 264.7 macrophages at various points in germination (germination progression indicated by increasingly darker shading). Data are representative of multiple, independent (3) biological replicates. (B) Phagocytosis of spores and/or yeast expressing GFP or mCherry (incubated individually or together) by RAW 264.7 macrophages in vitro. Results are illustrated as stacked bar graphs showing the mean±SEM percent of macrophages containing mCherry-positive fungal cells (red), GFP-positive fungal cells (green) or both (black) (n = 3) after 4 hours of co-incubation. Compositions of the populations incubated with macrophages are represented below the plots (G = GFP, m = mCherry). (C) Fungal burden quantity and composition in the lungs 3 days post-infection in mice infected with 5x10^6^ mCherry-spores, 5x10^6^ GFP-yeast, mCherry-yeast and GFP-yeast (5x10^6^ of each), or mCherry-spores and GFP-yeast (5x10^6^ of each). The results are expressed in jitter plots of pie-charts with the position of each pie chart indicating the fungal burden identified in a single mouse (y-axis). The contents of each pie-graph indicate the composition of the fungal burden in that same mouse (in 50 randomly-selected isolates per mouse). A horizontal line identifies the mean fungal burden of each group, and the mean composition of the fungi of all mice per group is represented by pie-graphs along the x-axis (n = 5–8). Compositions of the initial inocula are represented below the plot (G = GFP, m = mCherry). (D) Fungal burden quantity and composition in the lymph nodes (L.N.) 3 days post-infection in mice infected with mCherry-spores, GFP-yeast, mCherry-yeast and GFP-yeast, or mCherry-spores and GFP-yeast. Data are from lymph nodes from the same mice shown in (C) and are represented in the same manner. Empty pie charts represent mice in which no fungal colonies were recovered from the lymph nodes. For the difference in the ratios of mCherry+:GFP+ colonies in lung vs. lymph node p = 1.73x10^-8^ (by a Student's t-test).

To assess the kinetics of phagocytosis during germination, we carried out in vitro phagocytosis assays on populations of spores that were germinated for different lengths of time. Spores were incubated in rich growth medium for 0 to 16 hours, fixed with formaldehyde, stained with calcofluor white, and assessed in a fluorescence-based phagocytosis assay with RAW macrophages. We discovered that as germination progressed, the ability of macrophages to take up *Cryptococcus* decreased nearly linearly as a function of time (Fig 5A). This observation was also confirmed by visually quantifying phagocytosis of both formaldehyde- and UV-fixed spores in a time course of germination (S8 Fig). By 12 hours, spores had completed the morphological transition to yeast, and by 16 hours the population consisted of actively budding yeast. Cells from both 12 and 16 hours after the initiation of germination were phagocytosed at rates identical to live yeast controls, indicating that yeast resulting from germinated spores are indistinguishable from vegetatively growing yeast in terms of phagocytosis. These data also indicate that phagocytosis of spores diminishes over the course of germination, suggesting that the window for efficient phagocytosis of a germinating spore population is limited.

To determine whether the frequency of phagocytosis was strictly a feature of individual spores transitioning into yeast or whether spores and yeast were influencing the ability of phagocytes to take up other nearby cryptococcal cells (either spores or yeast), we carried out phagocytosis assays with mixed populations of spores and yeast. To track cryptococcal cells of each cell type separately, we generated spores and yeast expressing GFP or mCherry. RAW macrophages were then co-incubated with spores or yeast (expressing either GFP or mCherry) or 1:1 mixtures of both spores and yeast (expressing GFP and mCherry). Flow cytometry analysis was used to determine cryptococcal cell (GFP+ or mCherry+) association with live CD11b^+^ macrophages. As anticipated, we found that GFP-spores and mCherry-spores readily associated with macrophages. In contrast, macrophages incubated with GFP-yeast or mCherry-yeast showed no GFP+ or mCherry+ signals above background levels (Fig 5B). Macrophages co-incubated with the mixtures of spores and yeast did not show significant differences in the percentages of mCherry+ and GFP+ events compared to each cell type alone, indicating that spores and yeast do not influence phagocytosis of one another (Fig 5B). From these data we conclude that differences in spore and yeast phagocytosis result from intrinsic properties of the two cell types and are not caused by alterations (by either cell type) in the general phagocytosis competence of macrophages.

### Spores are preferentially trafficked to the lymph node in mixed infections with yeast in vivo

Based on the observations that spores are preferentially phagocytosed in vitro and that spore and yeast phagocytosis occur independently of one another in a mixed population, we hypothesized that spores would preferentially disseminate to the lymph node in a mixed infection in vivo. To test this hypothesis, we carried out mixed infections of spores and yeast using the mCherry+ and GFP+ strains described above. We then assessed the population composition recovered from the lungs and lymph node early in infection.

Mice were infected intranasally with 5x10^6^ mCherry-spores, 5x10^6^ GFP-yeast, 5x10^6^ mCherry-yeast + 5x10^6^ GFP-yeast, or 5x10^6^ mCherry-spores + 5x10^6^-GFP yeast. We deliberately infected mice with mCherry-yeast + GFP-yeast at twice the dose used for GFP-yeast alone to ensure that the total dose matched that of mCherry-spore + GFP-yeast infected mice. Thus, any differences in dissemination to the lymph node between yeast and spores reflects differences between the two cell types and not differences in dose. Three days after infection, the fungal burdens in the lungs and lymph nodes were quantified, and 50 colonies from each mouse were assessed for mCherry or GFP signals. We discovered that the average lung fungal burden in mCherry spore- and GFP yeast-infected mice was 2x10^5^ and 2x10^6^ CFUs, respectively (Fig 5C). Mice infected with mCherry-yeast + GFP-yeast contained an average lung fungal burden of 6x10^6^ CFUs, and mCherry-spore + GFP-yeast mice contained an average of 3x10^6^ CFUs. The higher lung fungal burdens in the groups of yeast-infected mice relative to spore-infected mice is unsurprising given that yeast are free to replicate from the onset of infection, whereas spores must undergo germination (an ~12 hour process) before they can replicate, leading to lower total CFUs in spore-infected mice.

The composition of the colonies from the lungs of all mice was as expected for the composition of the infectious inoculum. That is, mCherry spore-infected mice harbored 100% mCherry positive colonies, GFP yeast-infected mice harbored 100% GFP positive colonies, and mice infected with a 50:50 mixture of mCherry-yeast + GFP-yeast harbored an average of 47.6% mCherry positive colonies and 52.4% GFP positive colonies. Mice infected with a 50:50 mixture of mCherry-spores and GFP-yeast harbored an average of 14% mCherry positive colonies and 86% GFP positive colonies, consistent with the expected delay in replication of the products of mCherry-spores (Fig 5C).

Consistent with earlier dissemination results (Fig 4), spore-infected mice showed consistently higher fungal burdens in the lung-draining lymph nodes than yeast-infected mice. The lymph nodes of mCherry-spore-infected mice exhibited an average fungal burden of 75 CFUs, whereas all but one GFP-yeast-infected mouse contained no fungal cells in their lymph nodes (Fig 5D). Mice infected with mCherry-yeast + GFP-yeast averaged a lymph node burden of 58.6 CFUs, and mCherry-spore + GFP-yeast mice averaged 98.5 CFUs. Given our prior findings (Fig 4), the high fungal burden in the lymph nodes of the mCherry-yeast- + GFP-yeast-infected mice was unexpected; however, this value is heavily influenced by one lymph node that contained 198 colonies and is a significant outlier (as defined by a Grubbs’ outlier test, p < 0.05), likely due to contamination during dissection. Removal of this outlier decreases the average lymph node fungal burden of the mCherry-yeast + GFP-yeast group to 26, which is consistent with all other experiments.

Fungal composition in the lymph nodes reflected the inocula as one would expect for three of the four test groups; colonies from mice infected with mCherry-spores were 100% mCherry+, colonies from mice infected with GFP-yeast were 100% GFP+, and colonies from mice infected with a 50:50 mixture of mCherry-yeast:GFP-yeast were 46.6% mCherry+ and 54.4% GFP+. In stark contrast, lymph node colonies from mice infected with mCherry-spores and GFP-yeast were heavily skewed toward mCherry+ (82.5%) and away from GFP+ (17.5%) (Fig 5D). The high proportion of spore-derived cryptococcal cells (mCherry+) in the lymph nodes of these mice was particularly striking given the overrepresentation of yeast-derived cells present in their lungs (14% mCherry-spore-derived, 86% GFP-yeast-derived) (Fig 5C). The observed fungal composition (mCherry+:GFP+ ratio) between the lungs (14:86) and lymph nodes (82.5:17.5) was significantly different (p = 1.73x10^-8^), indicating a clear selection in vivo for spore-derived (mCherry+) colonies in the lymph node. From these data we conclude that intrinsic properties of spores determine their specific ability to traffic to the lung draining lymph node. These properties are non-transferrable to yeast and do not create a lung environment generally permissive for the escape of fungal cells.

### Spores are preferentially associated with alveolar macrophages in the mouse lung

Given that spores were phagocytosed efficiently in vitro and trafficked to the lymph node more readily than yeast, we hypothesized that spores preferentially associate with antigen-presenting phagocytes in the host lung. To test this hypothesis, we infected mice with spores or yeast and evaluated their relative association with dendritic cells (DCs) and alveolar macrophages (AMs) early in infection. Dendritic cells are canonical antigen presenting cells, and alveolar macrophages represent 90% of the sentinel immune phagocytes in a naïve, resting lung (and also have the ability to migrate for antigen presentation) [27]. A hallmark of both cell types is the presence of the surface integrin CD11c.

**Fig 6:**
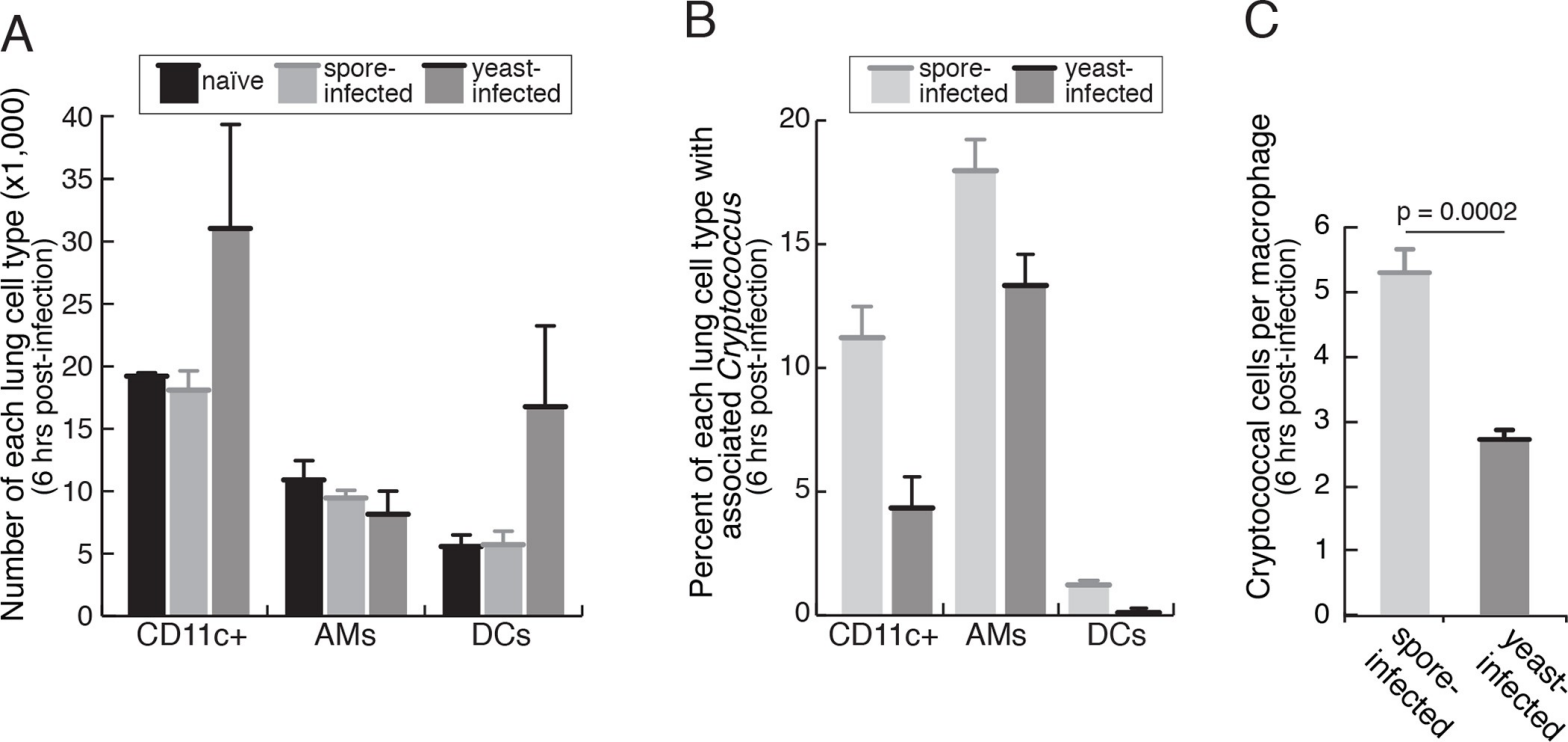
CD11c+ cells display more associated spores than yeast at 6 hours post-infection in the mouse lung. (A) The total number of each lung cell type (CD11c+, AMs = alveolar macrophages, DCs = dendritic cells) recovered at 6-hours post-infection in mice infected with PBS (“naïve," black bars, 2 mice), 5x10^6^ mCherry-spores (light gray bars, 5 mice), or 5x10^6^ mCherry yeast (dark gray bars, 5 mice). The results are expressed as bar plots illustrating the average ± SEM. For the number of DCs in spore- vs. yeast-infected mice p = 0.14. (B) Percent of each cell type (as identified in panel A) with associated mCherry+ *Cryptococcus* cells. The results are expressed as bar plots illustrating the average ± SEM. For the percentage of CD11c+ phagocytes associated with spores vs. yeast p = 0.019. (C) Cryptococcal cells per macrophage at 6 hours post-infection. Alveolar macrophages were recovered by bronchoalveolar lavage 6 hours post-infection in mice infected with 5x10^6^ mCherry-spores (light gray bars, 3 mice), or 5x10^6^ mCherry yeast (dark gray bars, 3 mice). The number of mCherry+ cryptococcal cells in 100 occupied macrophages was counted for each infected mouse. The results are expressed as the average occupancy of the three mice for each group ± SEM. For the number of spores vs. yeast per macrophage p = 0.0002 (by a Student's t-test).

Mice were infected intranasally with 5x10^6^ mCherry-spores, 5x10^6^ mCherry-yeast, or PBS as an uninfected control. Six hours after infections, whole lung homogenates were stained and analyzed by flow cytometry to quantify cryptococcal association with CD11c+ lung phagocytes (Fig 6 and S9 Fig). We first evaluated the total number of CD11c+ (live, CD45+CD11c+) cells in all groups (uninfected, spore-infected, and yeast-infected). We found that yeast-infected mice appeared to harbor more CD11c+ cells than uninfected and spore-infected mice. When we parsed CD11c+ cells into AMs (live, CD45+CD11c+SiglecF+) and DCs (live, CD45+CD11c+SiglecF-), we observed that the increased numbers of CD11c+ cells in yeast-infected mice were DCs (Fig 6A). This effect was consistent across independent experiments (S9 Fig) but given high variation in the number of DCs between animals, generated a significance value of p = 0.14 (Fig 6A). We then determined the percentage of each cell type (CD11c+, AM, and DC) that were mCherry+, indicating the association of at least one mCherry+ spore or yeast. We found that more CD11c+ cells were mCherry+ in the presence of spores than yeast (11.22% and 4.34% respectively), and this difference was robust (p = 0.002) and reproducible (p < 0.01 for all replicates), indicating that spores more readily associated with CD11c+ cells than yeast (Fig 6B and S9 Fig). When CD11c+ cells were further parsed in to AMs and DCs, we discovered that the increased spore association with CD11c+ cells was primarily with AMs. AMs were more likely to show spore association than yeast (18.0% and 13.3% respectively, p = 0.019), and this result was reproducible (p < 0.02 for all biological replicates) (Fig 6B and S9 Fig). In contrast, DCs consistently displayed an extremely low percent spore and yeast association overall (1.23% and 0.11% respectively) with very low numbers of overall mCherry+ events. Differences between spore and yeast association with DCs across experiments were highly variable with average p-values of p > 0.35 across biological replicates, suggesting that spores do not preferentially associate with DCs relative to yeast (Fig 6B and S9 Fig).

These cell association assays simply identified the presence or absence of mCherry+ signal for each CD11c+ phagocyte, so we could not discern the number of spores or yeast associated with each mCherry+ CD11c+ cell. To determine the ratio of cryptococcal spores or yeast to alveolar macrophages, we used direct microscopic visualization to count the number of spores or yeast per AM. Mice were infected with 5x10^6^ mCherry spores or yeast. Six hours post infection, alveolar macrophages were recovered by bronchoalveolar lavage, and the fungal loads of 100 mCherry+ phagocytes per mouse (n = 3) were counted. We found that on average, phagocytes from spore-infected mice contained two-fold more cryptococcal cells than yeast-infected mice (5.3 and 2.7 cells/phagocyte respectively) and that this difference was robust (p = 0.0002) (Fig 6C). These data indicate that the difference in association between spores and yeast with alveolar macrophages should be adjusted to account for differences in numbers of fungal cells per mCherry+ event, which effectively doubles the magnitude of the difference in favor of spore association.

Overall, from these data we conclude that early in infection, antigen presenting CD11c+ cells in the lung are at least 25% more likely to be associated with spores than yeast, those associations are primarily with alveolar macrophages (and not lung dendritic cells), and twice as many spores as yeast per phagocyte account for those associations.

### Dissemination of *Cryptococcus* spores to the lymph node is dependent on CD11c+ lung phagocytes

**Fig 7:**
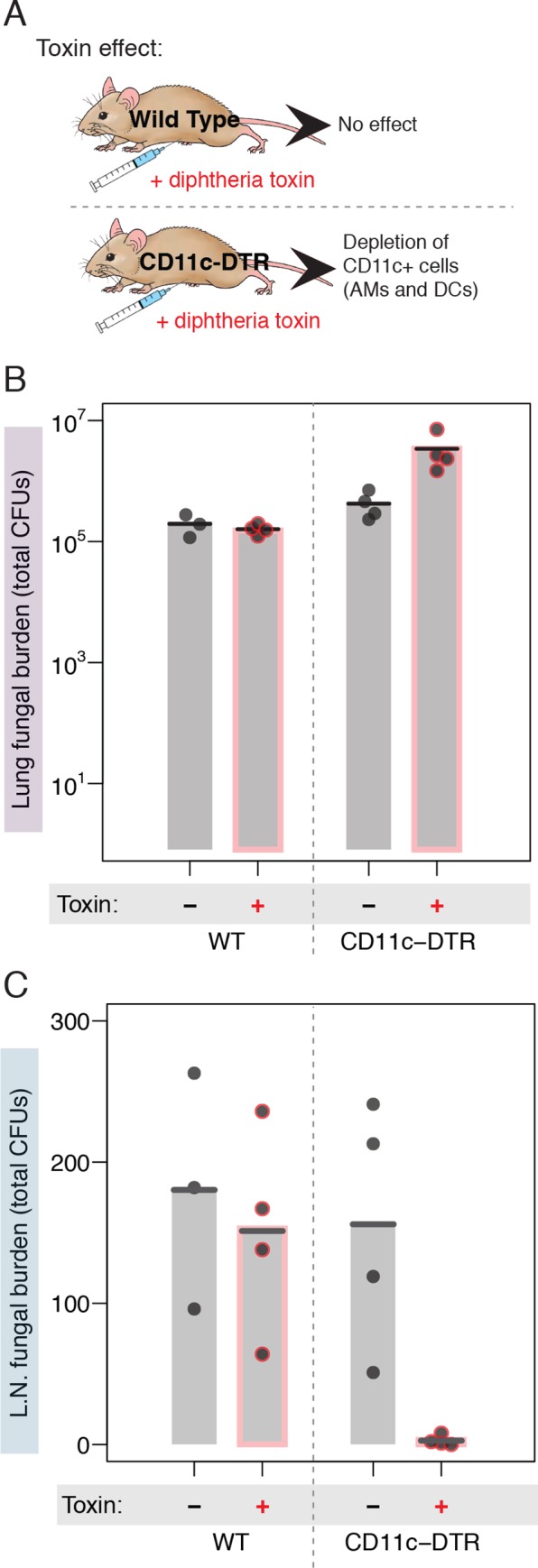
Dissemination of *Cryptococcus* spores to the lymph node is dependent on CD11c+ lung phagocytes. (A) Schematic illustrating the effects of administering diphtheria toxin to wild type (WT) mice or mice expressing the diphtheria toxin receptor under the control of a CD11c promoter (CD11c-DTR). AMs = Alveolar Macrophages, DCs = Dendritic Cells, DTR = Diphtheria Toxin Receptor. (B) Fungal burden (total colony forming units per organ) 2 days post-infection in the lungs of mice infected with 1x10^7^ spores derived from JEC20 x JEC21 crosses. Burdens are from wild type mice on the left and from CD11c-DTR mice on the right, each in the absence (gray bars) or presence (bars with red outline) of diphtheria toxin administration. (C) Fungal burden (total colony forming units per organ) 2 days post-infection in the lymph nodes of mice infected with 1x10^7^ spores derived from JEC20 x JEC21 crosses. Burdens are from wild type mice on the left and from CD11c-DTR mice on the right, each in the absence (gray bars) or presence (bars with red outline) of diphtheria toxin administration.

Increased CD11c+ phagocyte association and dissemination to the lymph node by spores provides strong support for a Trojan horse model of dissemination. Critical to this model is confirmation that dissemination is in fact CD11c+ phagocyte-dependent. We hypothesized that a reduction in the population of CD11c+ phagocytes would attenuate early dissemination to the lymph node by *Cryptococcus* spores. To test this hypothesis, we took advantage of transgenic CD11c-Diphtheria Toxin Receptor (CD11c-DTR) mice, which are modified to express the Diphtheria Toxin Receptor (DTR) under the CD11c promoter. This selective expression allows for transient depletion of CD11c+ cells (including lung DCs and AMs) upon diphtheria toxin (DT) administration (Fig 7A) [28]. Both WT and CD11c-DTR mice were treated with DT or PBS via intraperitoneal injection. After 36 hours, mice were infected intranasally with 1x10^7^ spores, and forty-eight hours post-infection the fungal burdens in lungs and lymph nodes were determined.

As expected, average total fungal burdens observed in the lungs of WT mice were very similar, whether or not they were treated with DT (2x10^5^ and 1.6x10^5^ CFUs, respectively). CD11c-DTR mice that were not treated with toxin also harbored lung burdens similar to WT mice (4.2x10^5^ CFUs) (Fig 7B). In contrast, there was a significant increase in CFUs in CD11c-DTR mice treated with toxin (3.4x10^6^ CFUs, p < 0.01 compared to WT) (Fig 7B), indicating that CD11c+ cells are likely important for stemming the proliferation of *Cryptococcus* and controlling early fungal growth following spore infections. Examining fungal burdens in mediastinal lymph nodes revealed that WT mice, WT mice treated with DT, and CD11c-DTR mice not treated with toxin showed no significant differences in fungal burden (WT = 180, WT + toxin = 151, CD11c-DTR = 156, all comparison p-values > 0.6) (Fig 7C). In contrast, the CD11c-DTR mice treated with DT showed a complete loss of dissemination to the mediastinal lymph nodes (average LN fungal burden = 2.75, p ≤ 0.01 compared to all other groups), despite the increased fungal lung burden in these same mice (Fig 7C). Thus, depletion of CD11c-expressing cells in mice resulted in the inability of *Cryptococcus* spores to reach the mediastinal lymph nodes. These data indicate that spores disseminate to the draining lymph node of the lung via a mechanism that is dependent on CD11c+ phagocytes.

**Fig 8:**
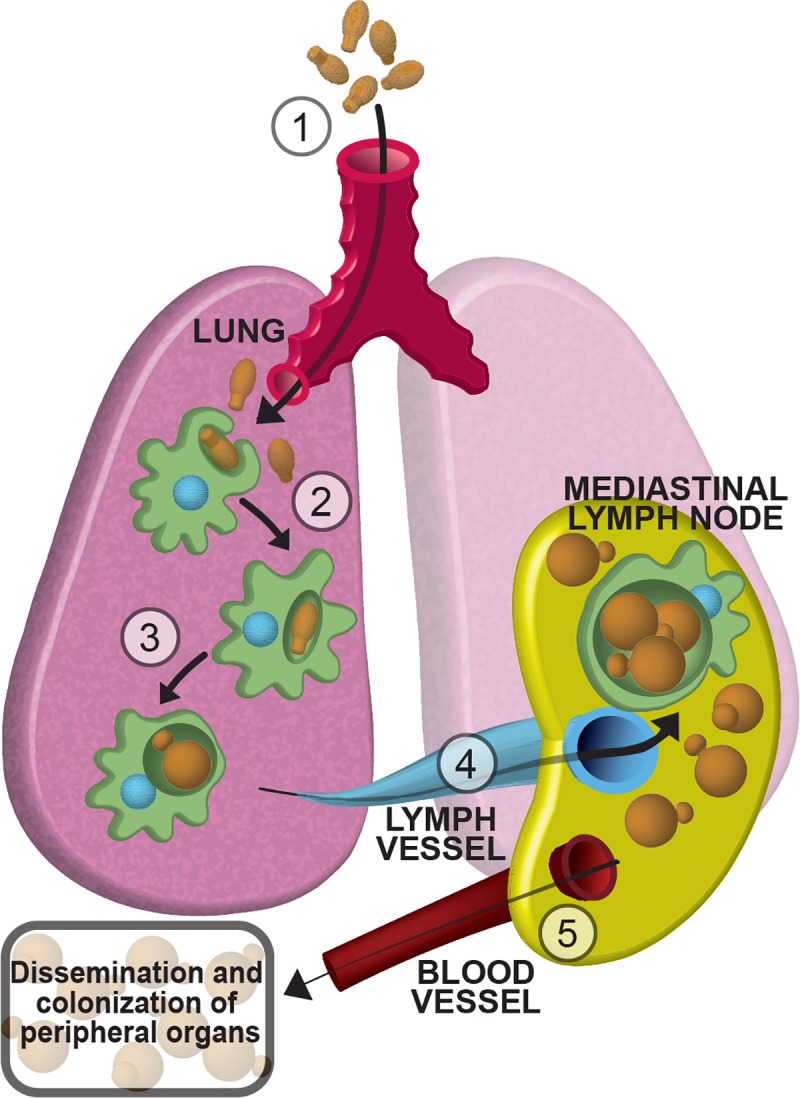
Model of cryptococcal spore-mediated disseminated disease. Graphical representation of our model of spore-mediated lung escape and disseminated disease: (1) spores are inhaled from the environment into the lungs where they are taken up by resident lung CD11c-positive phagocytes, likely alveolar macrophages (2). Inside phagocytes, spores are able to (3) germinate and efficiently (4) traffic to the mediastinal lymph nodes (the order of events 3 and 4 is unknown). From the lymph node, (5) *Cryptococcus* gains access to the blood stream, which can be used to disseminate to and colonize peripheral organs, eventually resulting in fatal CNS disease.

Based on these data, we propose model in which spores that infect the lung are phagocytosed by CD11c+ phagocytes (likely alveolar macrophages), survive inside those phagocytes, and are trafficked to the draining lymph node of the lung. Once they reach the lymph node, spores have access to the bloodstream, at which point they can access other tissues, including the brain (Fig 8).

## Discussion

Spores and yeast of *Cryptococcus* are likely natural infectious particles in human cryptococcosis and both have been shown to cause disease in a mouse model of infection [9,10]. However, the mechanisms by which *Cryptococcus* and other inhaled fungal pathogens escape the lung are poorly understood. Here, we evaluated the behaviors of spores and yeast from diverse strain backgrounds in a murine intranasal infection model to determine mechanisms of dissemination and disease. By comparing yeast- and spore-mediated infections, we discovered that disease outcomes can differ tremendously between infectious particles of the same organism, even when derived from the same strains. Most strikingly, using *Cryptococcus* yeast strains that typically do not cause disease in mice, we showed that spores derived from crosses between those avirulent yeast strains are fully virulent in mice and cause 100% fatal disease with symptoms reflecting meningoencephalitis. Thus, the nature of the infectious particle (yeast vs. spore) establishes the nature of the disease and its outcome (up to 75 days later). This finding is particularly intriguing because all visible spores in the mouse lung germinate into yeast within the first 18 hours after infection. As such, different relationships that spores and yeast establish with the host in the first day after infection confer differences in disease outcomes 2.5 months post-infection. These findings suggest that early interactions with the host innate immune response in the lung set the stage for the nature of disease.

This idea is consistent with our findings that mice infected with spores show earlier and higher rates of dissemination to the lung draining lymph node than mice infected with yeast, and these differences are dependent on CD11c-expressing resident lung cells. These data shed new light on the complicated relationship between resident immune cells of the lung (i.e. alveolar macrophages and dendritic cells) and infectious particles from a fungal pathogen. The critical roles of circulating lung phagocytes for protection against fungal infection in the lung have been clearly illustrated. In particular, depletion of phagocytes in the lungs of mammals results in rapid deterioration and death following infection with *Cryptococcus* [29]. However, our data also strongly suggest that at some frequency these immune cells that are normally tasked with defending against microbes can protect and chauffeur *Cryptococcus* out of the lung, leading to fatal disseminated disease. This type of Trojan horse mechanism of egress from the lungs has been observed for several bacterial pathogens, including *Bacillus anthracis* (via alveolar macrophages) and *Francisella tularensis* (via dendritic cells) [30,31]. The trafficking of *Cryptococcus* across the blood brain barrier has also been proposed to occur via phagocytes [8,32–34].

Here we present the first direct evidence of an inhaled fungal pathogen using a Trojan horse mechanism to escape the lung and cause disseminated disease (Fig 8). Most significant to this mechanism are our findings that 1) the efficiency of lymph node colonization in spore-infected mice is consistently orders of magnitude higher than in yeast-infected mice, 2) spores more readily associate with host lung phagocytes than yeast, and 3) trafficking of spores out of the lung is dependent on CD11c+ phagocytes. One prediction of this model is that the depletion of CD11c+ cells at the time of spore infections would lead to less dissemination to the bloodstream and consequently less dissemination to the brain, resulting in mitigation of disease or slower and/or lower fungal burdens in the brains. We made several attempts to test this prediction using the CD11c-DTR mice but discovered that CD11c-DTR mice treated with diphtheria toxin cannot tolerate subsequent infection with *Cryptococcus* spores or yeast. CD11c-DTR mice treated with even a single dose of diphtheria toxin and then infected with *Cryptococcus* experience uniform morbidity and mortality within 4 days, preventing longer-timeline experiments. As such, future experiments to test the long-term consequences of CD11c+ phagocyte depletion on spore dissemination from the lung to other tissues will require the development of a different system that imparts fewer pleiotropic effects.

Regardless of the events that occur after egress from the lung, our data strongly suggest a direct link between the frequency of spore phagocytosis by alveolar macrophages in the lung and the efficiency of dissemination in vivo. This finding is consistent with prior studies in which the relationship between phagocytosis efficiency and virulence was established with yeast. For example, one study showed that more virulent clinical (and laboratory) yeast strains are phagocytosed more efficiently in vitro than less virulent strains [35]. Similarly, it was observed that deletion of the gene encoding antiphagocytic protein 1 (*app1Δ*) in yeast increased phagocytosis by alveolar macrophages both in vitro and in vivo and increased disseminated disease in immunocompromised mice [36]. Assuming that phagocytosis efficiency and dissemination are linked directly in vivo, one possibility is that yeast employ the same mechanism to disseminate in the host as spores, but the mechanism for spores is significantly more efficient because of enhanced recognition and phagocytosis by immune cells.

Another (not mutually exclusive) possibility for increased lymph node colonization by spores is that intrinsic properties of spores imbue them with the ability to survive the host environment better than yeast, leading to more efficient dissemination from the lung. Spores are known to be more resistant than yeast to various forms of stress, including heat stress and reactive oxygen species [17], and this general resiliency of spores could extend to the harsh environment of a phagolysosome or similarly stressful intracellular environments. Better intracellular survival of spores (coupled with increased phagocytosis) may explain their higher efficiency of dissemination to the lung draining lymph node relative to yeast. Consequently, these differences in rates of survival and dissemination could ultimately lead to more cryptococcal cells reaching the blood brain barrier in spore-infected mice than yeast-infected mice, resulting in more opportunities for *Cryptococcus* to infect the brain and cause meningoencephalitis in spore-infected mice.

This model has major implications for the progression of mammalian cryptococcosis. Previous studies showed that environmental isolates of *Cryptococcus* were much less likely to cause disease in a mouse model of infection relative to clinical isolates (all strains were infected intranasally as yeast) [37]. Epidemiological data support the hypothesis that cryptococcosis in humans occurs after inhalation of *Cryptococcus* from environmental sources. If the majority of yeast strains isolated from the environment cannot cause disease (at least in mice), there is likely an alternative condition, particle, or selection that occurs in environmental sources that confers disease. Because *Cryptococcus* has a defined sexual cycle (through both opposite- and same-sex development), and population genetics studies of environmental isolates are consistent with sexual development occurring in nature, it follows that spores are likely to be produced in the environment. Given that spores are more resistant to environmental stressors than yeast, we hypothesize that spores harbor an enhanced capacity to cause disease (relative to their yeast parents). This was borne out by experiments in which spores derived from both highly virulent strains and avirulent strains showed advantages in virulence relative to yeast (e.g. higher brain CFUs at endpoint, more severe CNS symptoms, etc.). In fact, avirulent yeast parents gave rise to spores that caused fulminant disease in mice, lending credence to the idea that avirulent isolates (in the yeast form) might not be avirulent in the spore form. Such a finding could address the apparent paradox of how avirulent yeast isolates from the environment could be causing disease in humans. Thus far, studies of the spores of environmental isolates have been hampered by the fact that most are sterile or produce inviable spores [38,39]. Attempts to identify compatible mating partners for opposite-sex mating or stimulate same-sex mating of environmental isolates to levels required for robust spore isolation have not yet been successful. However, as more is learned about the signals that control sexual development and ultimately drive spore production, these technical challenges can be addressed.

Taken together, the data presented here support a specialized role for spores in causing mammalian disease that is dependent on association with host lung phagocytes. This relationship appears to occur with both yeast and spores, but spores can more effectively capitalize on their hosts to persist, escape the lung, and disseminate to other tissues to cause disease (Fig 8). Key in spore-mediated disease is the relationship between spore germination and survival in the host. If spores do not germinate, they do not cause disease, but remaining a spore long enough to capitalize on the lung innate immune response of the host appears to confer a significant advantage during infection. While the full relationship between the rate of spore germination and the host response to a transitioning particle remains to be determined, understanding the fundamental differences between spore- and yeast-mediated disease is a key part of elucidating the disease process. Finally, understanding the pathogenesis of spores of *Cryptococcus* promises to open new avenues into the development of novel therapeutics that could be effective in the prevention of fatal cryptococcosis and other diseases caused by the spores of invasive human fungal pathogens.

## Materials and methods

### Cell lines

*RAW 264*.*7 cells*. RAW 264.7 are adherent macrophages originally propagated from an Ableson murine leukemia virus-induced tumor in a male BALB/c mouse. Cells were cultured in RPMI 1640 + 10% FBS at 37°C and 5% CO_2_. Cells were harvested using a cell scraper and passaged every 2–3 days upon reaching 80–90% confluency with a 1:5–1:10 split ratio.

*JAWSII cells*. JAWSII cells are a dendritic-like mix of adherent and suspended cells originally derived from the bone marrow of a C57Bl/6 mouse (sex unknown). Cells were cultured at 37°C and 5% CO_2_ in alpha-MEM (with L-glutamine) + 1mM sodium pyruvate + 5 ng/mL GM-CSF + 20% FBS. Cells were subcultured every 1–2 weeks by removing attached cells with rinsing with PBS, and incubating with 0.25% Trypsin + EDTA for 5 minutes at 37°C and splitting at a 1:2 ratio.

### Strain handling

All *Cryptococcus* strains were handled using standard techniques and media as described previously [40]. *C*. *neoformans var*. *grubii* strain H99 (serotype A, mating type α), *C*. *neoformans var*. *grubii* strain BT63 (serotype D, mating type **a**), *C*. *neoformans var*. *neoformans* strain JEC20 (serotype D, mating type **a**), *C*. *neoformans var*. *neoformans* strain JEC21 (serotype D, mating type α), *C*. *neoformans var*. *neoformans* strain B-3502 (serotype D, mating type **a**), and *C*. *neoformans var*. *neoformans* strain B-3501 (serotype D, mating type α) were grown on yeast extract peptone dextrose (YPD) agar plates at 30°C and stored at 4°C. All experiments conducted with *C*. *neoformans* yeast cells employed a 1:1 mixture of **a**:α mating type unless indicated otherwise.

### Spore isolation

*Cryptococcus* spores were created and purified as described previously [17]. Briefly, yeast of both mating types (BT63 (**a**) with H99 (α), B-3502 (**a**) with B-3501 (α), JEC20 (**a**) with JEC21 (α)) were grown separately on YPD agar for 2 days and then mixed in equal parts in phosphate buffered saline (PBS). This suspension was then spotted onto aged V8 agar (pH 7.0) and grown for 4–5 days at room temperature to facilitate **a** x α sexual development and the production of spores. Resulting crosses were then scraped off the plate, resuspended in a Falcon tube containing 65% Percoll/1X PBS, and subjected to gradient centrifugation at 10°C for 20 minutes and 4000 RPM in a swinging bucket tabletop centrifuge. The resulting spores were collected from the bottom of the Falcon tube using a 20G needle. Purity and total number of spores were determined by counting directly on a hemocytometer and plating to YPD to confirm the concentration of viable spores.

### GFP/mcherry strain creation

For experiments to track and discern spore and spore-derived cryptococcal cells from yeast and yeast-derived cryptococcal cells we created two mating strain pairs expressing GFP-NAT^R^ or mCherry-NEO^R^ in the JEC20 and JEC21 background. In both cases expression was driven by the histone H3 promoter, and the fluorescent protein contained a nuclear localization signal (NLS) from Nhp6b02 (CNE04220). To ensure that the integration of the expression cassette was consistent between strains, and that it did not interfere with any genes, the expression cassette was integrated at the site homologous to the “safe-haven” site previously identified in H99 [41]. In JEC21 this intergenic site landed between the genes CNA07550 and CNA07560, and this site was targeted via homologous recombination. The left flank and right flank for homologous recombination were obtained from genomic DNA isolated from JEC21 using PCR. The resulting left flank was 756 bp in length and was created using primers CHO5360 and CHO536 (primers listed in S1 Table), the right flank was 775 bp in length and was created using primers CHO5362 and CHO5363. Using Gibson cloning, the flanks were then added to the previously created plasmid pCH1227 (digested with Apa1 and HindIII, and fragments purified following gel extraction) (thanks to JM Davis). The resulting plasmid (pCH1351) was sequence-verified and encodes a histone H3-driven enhanced green fluorescent protein (EGFP) with an 80-amino acid NLS and nourseothricin resistance (NAT^R^) cassette with flanks for homologous recombination into JEC21. pCH1351 was digested with SalI and introduced into JEC21 using biolistic transformation to create strain CHY3952. Integration of the GFP expression cassette at the intended safe-haven locus was confirmed using PCR with primers annealing to GFP and to regions upstream or downstream of the flanks used for homologous recombination (primers CHO5382 and CHO5383). CHY3952 was then crossed with JEC20 to generate a congenic GFP-NLS-expressing **a** strain (CHY3955). An identical strain pair expressing mCherry and G418^R^ was created by replacing the GFP-NAT^R^ cassette with and mCherry-G418^R^ cassette via homologous recombination in strain CHY3952 (Primers listed in S2 Table) and then backcrossing with JEC20. From this backcross, strains CHY4028 (α) and CHY4031 (**a**), were isolated for use as a mating pair. All GFP and mCherry strains were confirmed to harbor a single integration of the H3-NLS-fluor-marker cassettes at the desired locus.

### Murine model of infection

The virulence, dissemination, and histology of *Cryptococcus* were determined using age-matched male and/or female mice (between 6 and 12 weeks) in the C57Bl/6 genetic background (from Jackson Laboratory). CD11c-DTR mice were bred in-house under specific pathogen-free (SPF conditions). Mice were housed and handled under appropriate guidelines in accordance with our IACUC-approved protocol #A005118-R01. Numbers of mice needed for robust statistical outcomes were determined using an a priori power analyses as appropriate.

For intranasal instillation, the indicated numbers of spores or yeast in a volume of 50 μl PBS were applied to the nares of anesthetized mice as described previously [10]. Direct blood stream infections were carried out by administration of 1x10^6^ yeast or spores in 250μl PBS via injection into the tail vein. Mice were monitored twice daily, and euthanized if they appeared moribund (i.e. exhibited hunched behavior, ruffed fur, difficulty walking, and/or >20% weight loss). Moribund mice were euthanized via CO_2_ asphyxiation, according to AMVA guidelines. Experiments assessing survival times were terminated once all mice succumbed to cryptococcosis or survived 100 days post-infection. Replicate data: For H99/BT63 survival curves (see Fig 1A), a second biological replicate was carried out with 4 mice per group, also generating no significant differences among groups. For H99/BT63 CFU analyses (see Fig 1B), a second biological replicate with 3 mice per group was carried out, also generating significant differences between CFUs in the brain (p = 0.018) but not the lungs (p = 0.30) or other tissues, including spleen, liver, kidney, and blood (all p-values ≥ 0.1). In all pairwise comparisons, significance values were generated using a two-sample, two-tailed Student's t-test.

For B-3501/B-3502 survival curves (see Fig 2) in which yeast-infected mice did not show any symptoms, mean time to death was assigned at 100 days post-infection. A second biological replicate of the B-3501/B-3502 spore vs. yeast survival experiment also resulted in a significant difference in time to death between spore- and yeast- infected mice (p = 2.7x10^-3^ by a Log-Rank test for Kaplan-Meier survival analysis) [42]. Panels 2B and 2C are representative of two independent biological replicates of fungal loads in organs.

### Early dissemination analysis

Dissemination of fungal burden in spore and yeast-infected mice was assessed by CFU determination in mouse organs as described previously [10]. Briefly, mice were euthanized at indicated time points, and organs (including lungs, kidney, brain and one of the mediastinal lymph nodes) were surgically removed, weighed, and homogenized by bead beating in sterile PBS. Tissue homogenates were serially diluted and plated on YPD agar. Colony forming units per gram of tissue were then determined after 2–3 days of growth at 30°C. For experiments determining the mCherry-NEO and GFP-NAT composition of the fungal burden, up to 50 colonies per organ per mouse were randomly selected, patched to fresh YPD plates, replica-plated to YPD-NAT and YPD-NEO agar and assessed for growth.

### Histology

For histological analysis, C57Bl/6 mice were infected with 2.5x105 B-3501 x B-3502 spores or their parental yeast strains. At days 3, 6, 14, 21 and endpoint (morbidity for spore-infected or 100 days for yeast-infected), 3 spore and 3 yeast-infected mice were sacrificed, and their lungs and brains were fixed in formalin. Cross sections of each organ were prepared, mounted, and stained with Mucicarmine by the histology lab at the UW School of Veterinary Medicine. Slides were imaged at 100X magnification with a Zeiss Axioskop 2 Plus microscope.

### Lung immune response characterization

#### Lung leukocyte isolation

Lung leukocytes were isolated as previously described [42–45] with modification. Lungs from 4 mice per group were separately collected in 2mL digestion buffer (RPMI with 1% fetal calf serum, 1 mg/ml collagenase D, 100units/ml penicillin, 100μg/mL DNAse) and homogenized by pressing through a 70μm filter. Homogenates were incubated for 30 minutes at 37°C, and RBCs were subsequently lysed using ACK lysis buffer, followed by washing. Total number of the cells in the suspension was then determined by counting using a hemocytometer.

#### Flow cytometry surface staining

A protocol of surface staining for flow cytometric analysis was carried out as described previously [43,44,46,47] with minor modifications. Single cell suspensions were first stained with 1:250 fixable Live/Dead (near IR), then incubated with unlabeled FcRIII/II to block Fc receptors and subsequently stained with 1:200 dilutions of fluorescently labeled antibodies (CD45, SiglecF, CD11b, CD11c, Ly6G, Ly6C, MHCII) for 30 minutes at 4°C, resuspended in 2% paraformaldehyde and stored at 4°C protected from light until acquisition.

#### Ex vivo stimulation and intracellular cytokine staining

Ex vivo stimulation and cytokine staining was performed as described previously [44,45,48]. Briefly, cells were stimulated for 5 hours in the presence of Golgi Stop at 37°C with 0.1 μg/ml anti-CD3 antibody and 1 μg/ml anti-CD28 antibody. Cells were subsequently stained for surface markers as described above (CD45, CD4, CD44), permeabilized using the BD Cytofix/Cytoperm kit, stained for intracellular antibodies (IFNgamma, IL-13, IL-5), and resuspended in 2% paraformaldehyde and stored at 4°C protected from light until acquisition.

### Colony forming unit (CFU)-based phagocytosis assays

To analyze the phagocytosis of live *Cryptococcus* spores and yeast by bone marrow-derived macrophages, RAW264.7 macrophages, and JAWSII dendritic like-cells, a CFU fungal association assay was used as described previously [24]. Bone marrow-derived macrophages were obtained from the femurs of wild type C57BL/6. Femurs were flushed with 5 mL cold PBS and passed through a 70 μm filter. Red blood cells (RBCs) were lysed using ACK lysing buffer, the remaining cells washed, plated with 2x10^6^ per petri dish in DMEM + 10% FBS with 3 ng/mL recombinant GM-CSF. On day 3, the medium was refreshed, and on day 7 the cells were harvested with trypsin for in vitro assays. Phagocytes were co-cultured with spores or yeast for 4 hours at an MOI of 10:1. *Cryptococcus* that had not adhered or been phagocytosed was then removed by washing 3 times with PBS. Macrophages were lysed with 0.01% Triton X-100 (a concentration known not to affect the viability of *Cryptococcus*) to release intracellular *Cryptococcus*; the lysate was serially diluted and plated on YPD to determine the number of macrophage-associated *Cryptococcus* cells. After 3 days of growth at 30°C, colony forming units were counted, and the percentage of macrophage association was calculated as (# CFUs from lysate)/(CFUs introduced).

### In vivo germination assay

To determine the efficiency of spore germination in a mouse lung, three mice were infected intranasally with JEC20 x JEC21 spores (3 mice) and as a control, one mouse was infected with JEC20 + JEC21 yeast. Eighteen hours post-infection, the mice were sacrificed and cells in the mouse lung were collected by bronchoalveolar lavage and fixed in 4% formaldehyde. Cells were then observed by microscopy on a Zeiss Axioskop 2 Plus microscope at 10000X magnification. Approximately 20 cryptococcal cells per mouse were randomly chosen, and the width and length of each was measured and used to calculate their aspect ratios (width/length). Lower aspect ratios indicated earlier germination states, while aspect ratios near 1 indicated fully germinated, spherical yeast. Dormant spores in PBS alone and vegetatively growing yeast collected from YPD medium were used as controls.

### Fluorescence-based phagocytosis assay

To assess differences in uptake of partially germinated *Cryptococcus* spores compared to growing yeast cells, an adapted fluorescence-based assay was used [49]. RAW 264.7 cells were seeded into 96 well plates with 3x10^4^ macrophages per well in 100μL per well in RPMI + 10% FBS and allowed to adhere overnight. *Cryptococcus* spores were allowed to germinate for 0, 0.5, 1, 2, 4, 8, 12 and 16 hours in YPD shaking at 30°C alongside vegetatively growing yeast. Fungal cells were fixed overnight at 4°C in 4% formaldehyde, stained with 50μM calcofluor white for 5 minutes, washed three times with PBS and counted using a hemacytometer. Medium was removed from the macrophages, and for each condition fungal cells were introduced to macrophages at an MOI of 100:1 (3x10^6^ fungal cells/well) in RPMI + FBS. A serial dilution control curve (1:2 dilutions from 3x10^6^ to 9.4x10^4^ cells) was created for each condition. After a phagocytosis period of four hours, fluorescence of each well was measured using a plate reader, providing a value corresponding to the number of fluorescently labeled *Cryptococcus* cells in each well. Then extracellular (non-phagoctyosed) cell fluorescence was quenched with the addition of 100μl trypan blue, and fluorescence was measured again (corresponding to the number of phagocytosed cells). Wells containing macrophages fixed prior to phagocytosis were included as a background fluorescence/no phagocytosis control. The number of cells phagocytosed for each germinated population was determined by using the best-fit line equation for each of the standard curves generated. Percent phagocytosis was calculated as [100 x (# of cells phagocytosed/number of cells introduced)].

### Direct visualization phagocytosis and association assay

RAW 264.7 cells were plated on glass slides in a 24 well plate with 3x10^5^ cells per well. JEC20 x JEC21 spores were germinated for different lengths of time (0, 0.5, 2, 4, 8, 12, 24 hours) in YPD shaking at 30°C, at which point they were washed three times with PBS and fixed (either by UV irradiation or in 4% formaldehyde), stained with 50μM calcofluor white for 5 minutes, washed three times with PBS and counted using a hemacytometer. A live spore and yeast control were included. Medium was removed from RAW 264.7 cells and replaced with 1mL of medium containing stained fungal cells at an MOI of 1:1. Phagocytosis was allowed to progress for 4 hours, wells were washed 3 times with PBS, and glass slides were then fixed and mounted. For each condition, 10 randomly selected fields were observed with epifluorescence on a Zeiss Axioskop 2 Plus microscope at 630X magnification. The number of fungal cells bound to and internalized by macrophages was counted to determine the total number of fungal cells associated at each condition. This association was normalized to the 0 hr time point. The average extent of germination of the cells introduced, bound, and internalized was calculated for each condition as described in the methods for germination assays.

### Flow cytometry phagocytosis assay

Independent and mixed phagocytosis assays of mCherry-NEO and GFP-NAT spores and yeast by RAW 264.7 cells were assessed by flow cytometry. RAW 264.7 cells were seeded in 96 well plates with 1x10^5^ cells per well in RPMI + 10% FBS and allowed to adhere overnight. mCherry spores, GFP spores, mCherry yeast or GFP yeast were introduced to macrophages at an MOI of 5:1. To assess how the cryptococcal cell types influenced phagocytosis of one another, spores and yeast with alternate fluors were added together and introduced to macrophages, resulting in a final MOI of 10:1. After 4 hours, macrophages were washed 3X with PBS to remove any unassociated cryptococcal cells. Macrophages were lifted from the wells by 10 minutes of incubation with Versene at 37°C and vigorous pipetting. Cells were washed once with RPMI + 10% FBS and then stained with a 1:200 dilution of anti-CD11b conjugated to PE-Cy7 and 1:1000 fixable Live/Dead yellow. Association of mCherry+ and GFP+ fungal cells with live macrophages (CD11b+L/D-) was quantified using flow cytometry on a BD LSRII cytometer.

### In vivo association of *cryptococcus* with CD11c+ lung phagocytes

Mice were infected intranasally with 5x10^6^ spores or yeast expressing mCherry (5 mice each) or a PBS control (2 mice). Six hours post-infection, mice were euthanized by asphyxiation with CO_2_, and whole lungs were recovered. Lung leukocyte isolation and surface staining for analysis by flow cytometry was carried out as described above. Live leukocytes (L/D-, CD45+) were identified and further gated to identify alveolar macrophages (CD11c+, SiglecF+) and lung dendritic cells (CD11c+, SiglecF-). Association of cryptococcal cells with each of these subsets was determined by positive mCherry signal.

In a separate experiment, the number of cryptococcal cells per phagocyte was determined microscopically. Mice were infected intranasally with 5x10^6^ spores or yeast expressing mCherry (3 mice per cell type), and lung cells were recovered by bronchoalveolar lavage at 6 hours post infection. Red blood cells were lysed, and the samples were fixed and examined using epifluorescence on a Zeiss Axioskop 2 plus microscope at 1000X magnification. The number of mCherry+ cryptococcal cells inside 100 fungus-containing phagocytes per mouse was counted.

### CD11c+ cell depletion and spore dissemination

CD11c-Diphtheria Toxin Receptor (CD11c DTR) mice were purchased from the Jackson Laboratory and bred in-house. WT C57BL/6 mice or CD11c-DTR mice were administered 500μL of 20ng/mL diphtheria toxin by intraperitoneal injection (alongside non-treated controls). Depletion was allowed to progress for 36 hours, at which time mice were infected intranasally with 1x10^7^ JEC20 x 21 spores. Two days post infection, mice were sacrificed, and their lungs and lymph nodes were homogenized and plated onto YPD to assess fungal burden through counting CFUs as described above.

### Ethics statement

All experiments were carried out in accordance with the recommendations in the Guide for the Care and Use of Laboratory Animals of the National Institutes of Health under University of Wisconsin-Madison Institutional Animal Care and Use Committee-approved protocol number A005118. UW-Madison is an AAALAC accredited institution.

## Supporting information

S1 Fig Histological analysis with mucicarmine staining of brains and lungs taken from mice infected with (A) 2.5x10^5^ yeast as a 1:1 mixture of B-3502 + B-3501, or (B) 2.5x10^5^ spores derived from a cross between B-3502 and B-3501.
(PNG)

S2 Fig Average lung fungal burden in total lung homogenates recovered 30 minutes after infection with 5x10^5^ yeast as a 1:1 mixture of B-3502 + B-3501 or 5x10^5^ spores derived from a cross between B-3502 and B-3501 as a percentage of the initial inoculum (n = 3 mice per group).
(TIF)

S3 Fig Quantification of (A) total lung cells as counted by hemacytometer, or (B) total leukocytes (CD45+ as assessed by flow cytometry) elicited by intranasal infection with 2.5x10^5^ yeast of a 1:1 mixture of B-3502 + B-3501, 2.5x10^5^ spores from a B-3502 x B-3501 cross, or 1x10^5^ H99 yeast at 11 days post infection. The Y-axis indicates total number of host cells, and error bars represent the mean ± standard error of the mean (SEM).
(TIF)

S4 Fig Association of JEC20 x JEC21 spores (orange) or JEC20 + JEC21 yeast (blue) after 4 hours of co-incubation with RAW 264.7 cells (left panel), JAWS II cells (middle panel), or bone marrow-derived macrophages (right panel) at an MOI of 10:1. (Assessed using CFU-based phagocytosis assays).
(TIF)

S5 Fig Total fungal burden (colony forming units) in the spleen and blood of mice (3 per group) infected intranasally with 1x10^6^ yeast in a 1:1 mixture of JEC20 + JEC21 (red) or 1x10^6^ spores derived from a JEC20 x JEC21 cross (blue) after 1, 5, and 7 days of infection.
(TIF)

S6 Fig Lung and mediastinal lymph node fungal burdens early in infection for mice infected with spores (blue) or yeast (red) from various strains and inocula of *Cryptococcus* (as indicated above figure panels for A and B). Panel C used GFP+ derivatives of JEC20 and JEC21 (strains CHY3955 and CHY3952).
(PNG)

S7 Fig Germination state of cryptococcal cells recovered from spore- or yeast-infected mice 18 hours post-infection compared to the measured germination state of spore and yeast controls in vitro. Bars show average value of 20 cryptococcal cells measured per control or per infected mouse (n = 3 mice for spore-infected). Error bars show SEM, and p-values were calculated using a Student’s t-test.
(TIF)

S8 Fig Relationship between germination state of cryptococcal cells and association (adherence and phagocytosis) with RAW 264.7 macrophages. Cryptococcal cells were fixed using (A) formaldehyde or (B) UV at various time-points during germination, and adherence and phagocytosis were assessed microscopically.
(TIF)

S9 Fig Biological replicates showing that CD11c+ cells display increased association with cryptococcal spores compared to yeast at 6 hours post-infection in the mouse lung.
(TIF)

S1 Table Primers used for cloning pCH1351. (DOCX)

S2 Table Primers used for the creation of strains CHY4028 and CHY4031. (DOCX)
