# Supplementary figures and images for: Infectious particle identity determines dissemination and disease outcome for the inhaled human fungal pathogen Cryptococcus
Source: PLoS Pathog. 2019 Jun 27;15(6):e1007777. doi: 10.1371/journal.ppat.1007777 (PMC6597114; doi:10.1371/journal.ppat.1007777)

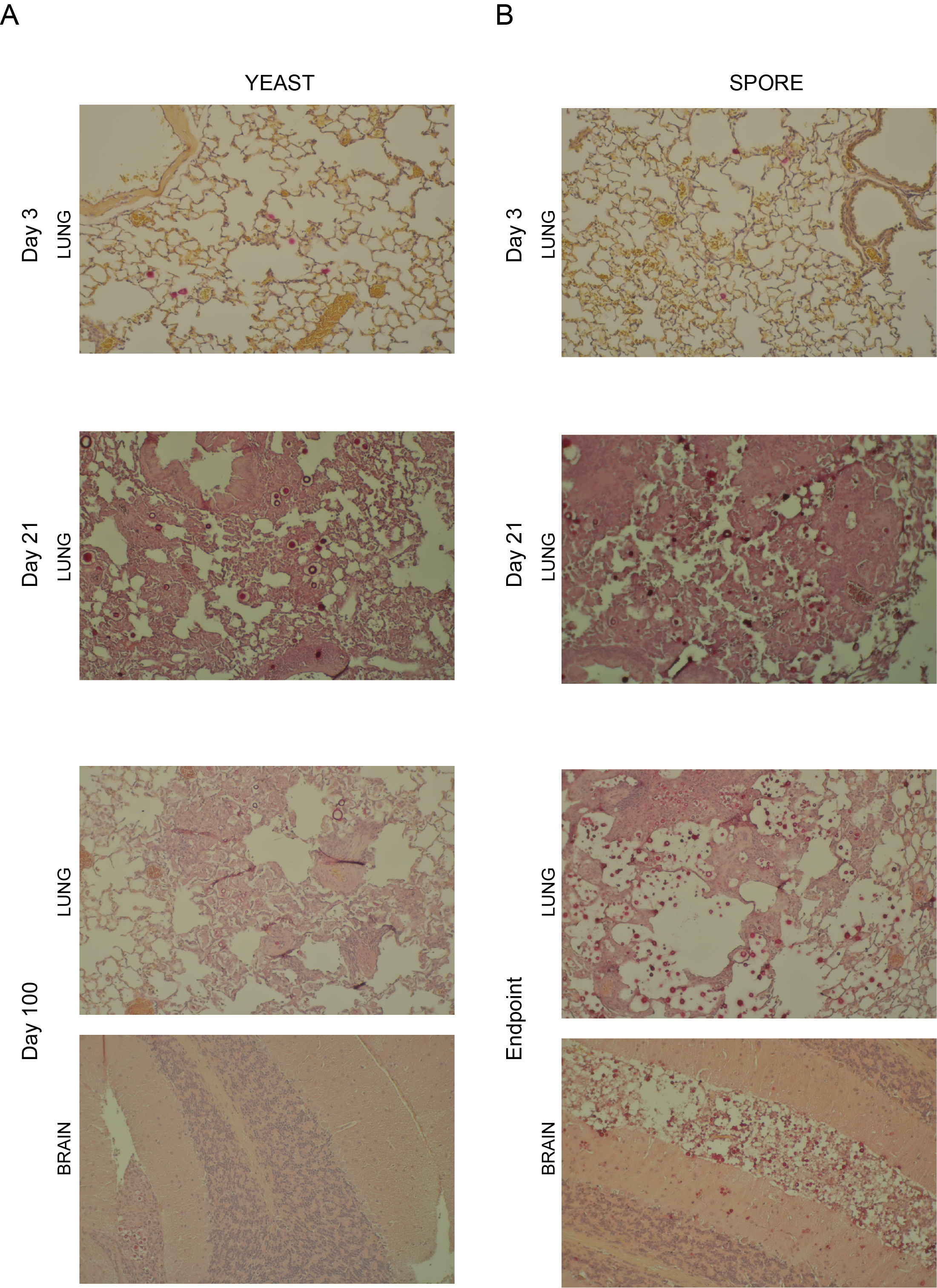

Supplement: S1 Fig — Histological analysis with mucicarmine staining of brains and lungs taken from mice infected with (A) 2.5x10^5^ yeast as a 1:1 mixture of B-3502 + B-3501, or (B) 2.5x10^5^ spores derived from a cross between B-3502 and B-3501. (PNG) [file ppat.1007777.s001.png]

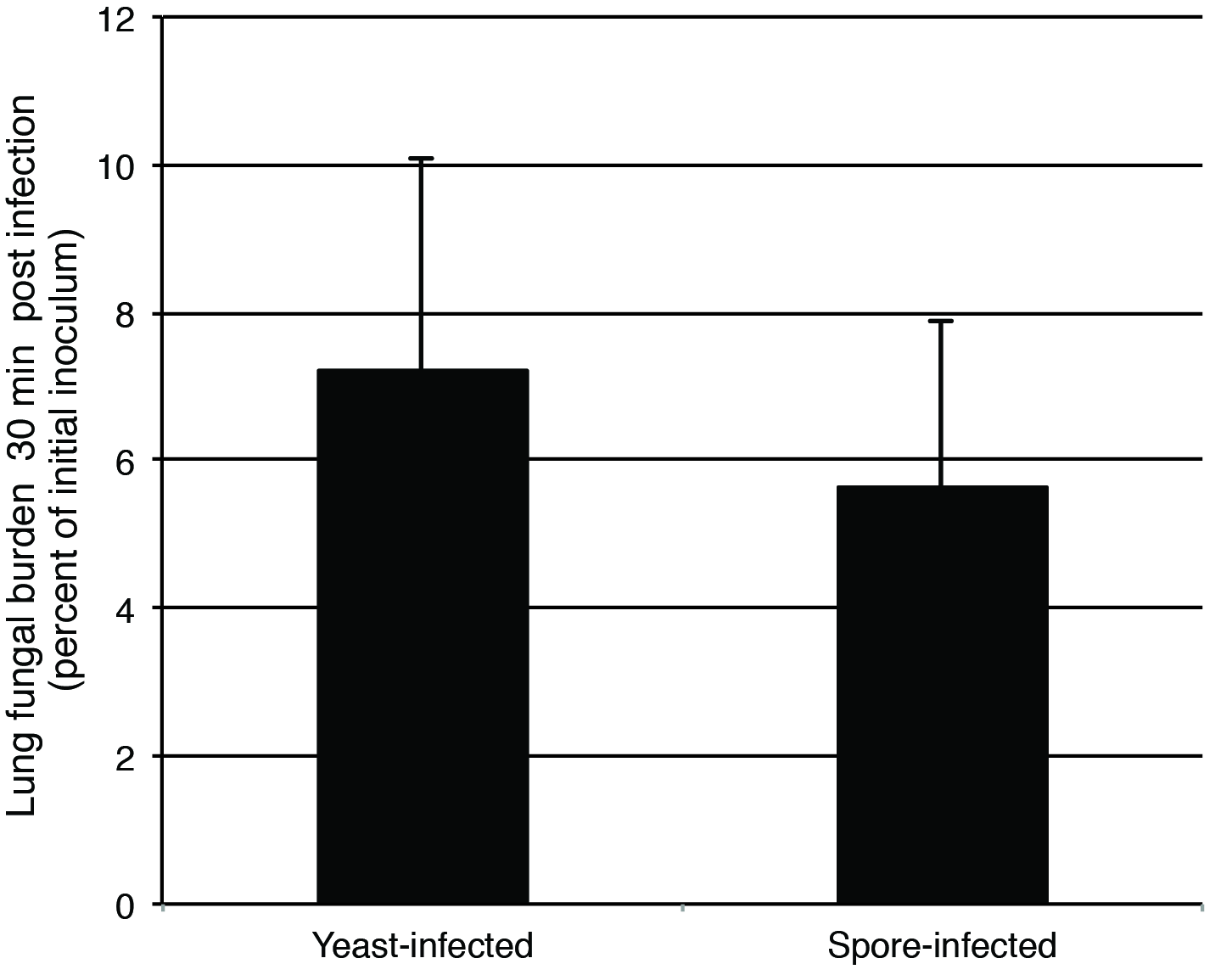

Supplement: S2 Fig — Average lung fungal burden in total lung homogenates recovered 30 minutes after infection with 5x10^5^ yeast as a 1:1 mixture of B-3502 + B-3501 or 5x10^5^ spores derived from a cross between B-3502 and B-3501 as a percentage of the initial inoculum (n = 3 mice per group). (TIF) [file ppat.1007777.s002.tif]

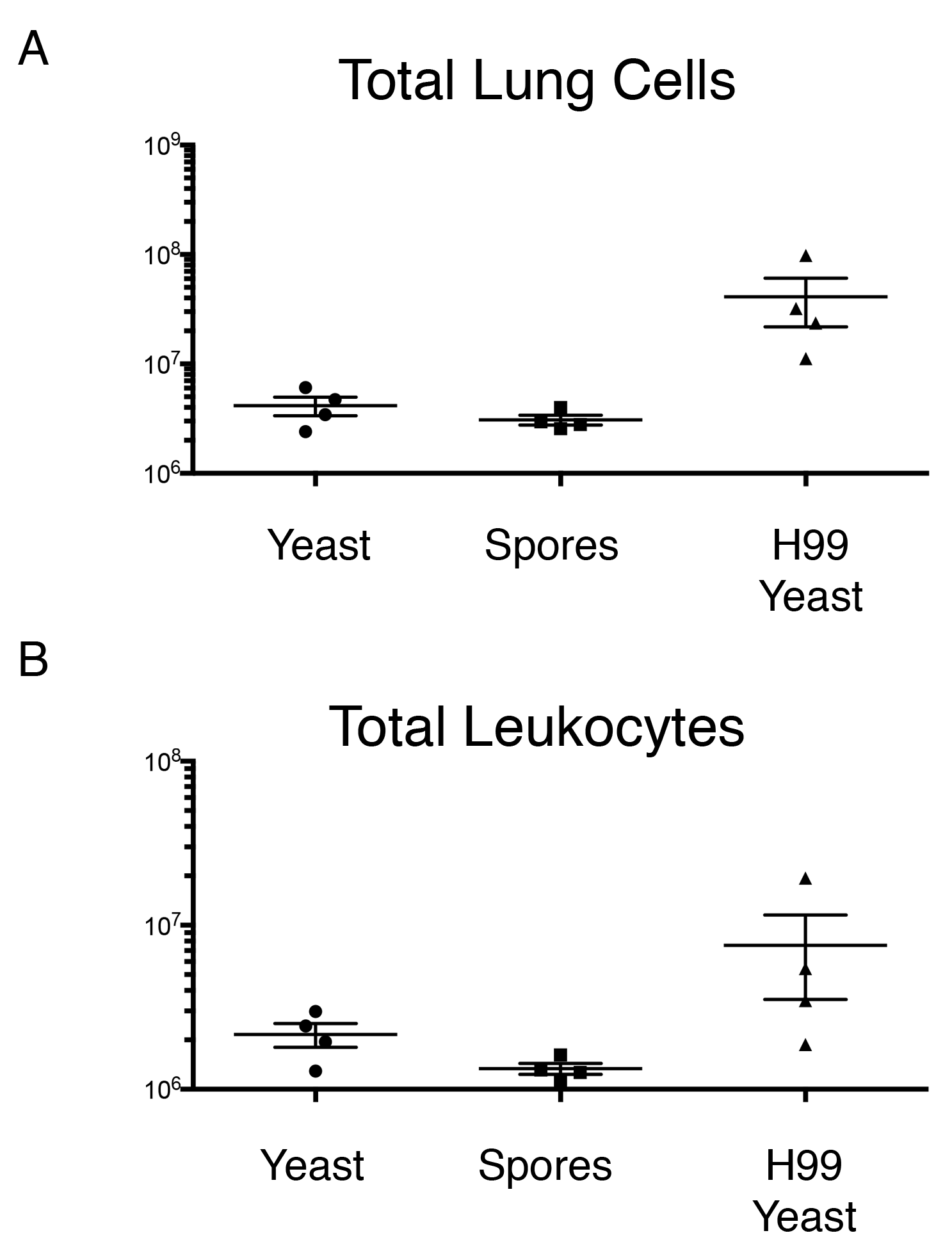

Supplement: S3 Fig — Quantification of (A) total lung cells as counted by hemacytometer, or (B) total leukocytes (CD45+ as assessed by flow cytometry) elicited by intranasal infection with 2.5x10^5^ yeast of a 1:1 mixture of B-3502 + B-3501, 2.5x10^5^ spores from a B-3502 x B-3501 cross, or 1x10^5^ H99 yeast at 11 days post infection. The Y-axis indicates total number of host cells, and error bars represent the mean ± standard error of the mean (SEM). (TIF) [file ppat.1007777.s003.tif]

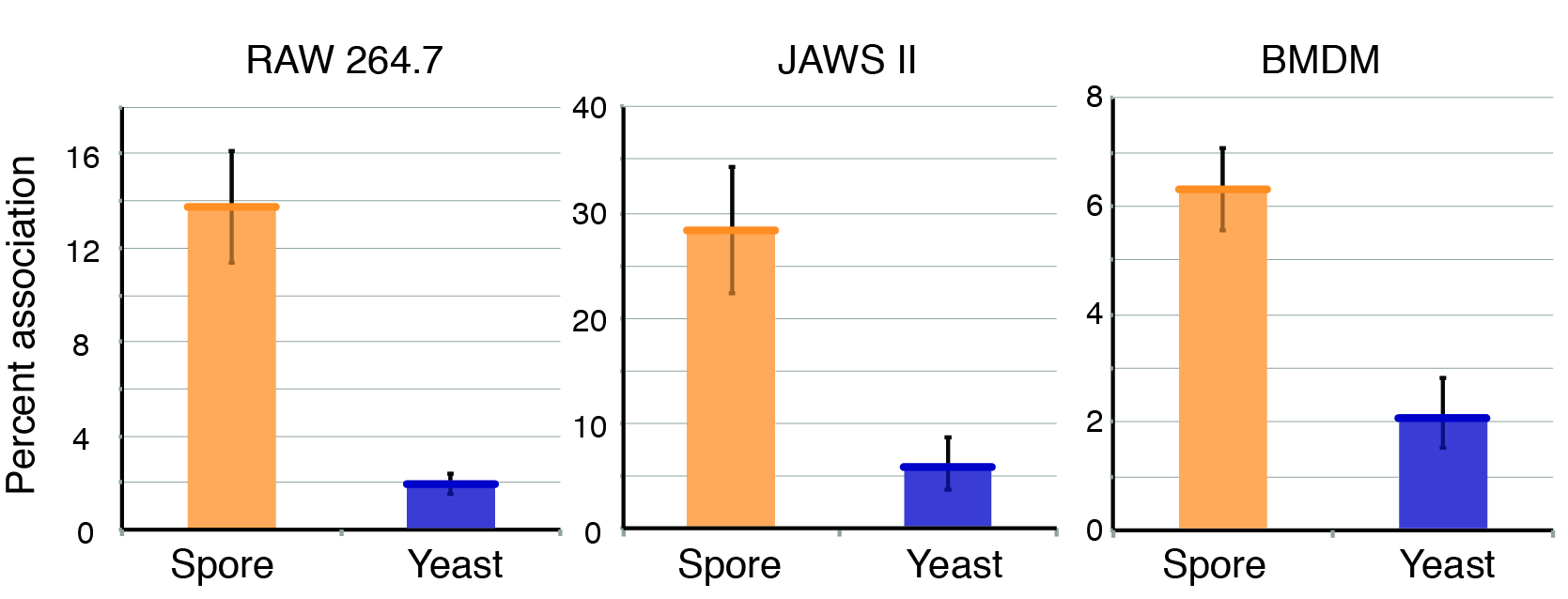

Supplement: S4 Fig — Association of JEC20 x JEC21 spores (orange) or JEC20 + JEC21 yeast (blue) after 4 hours of co-incubation with RAW 264.7 cells (left panel), JAWS II cells (middle panel), or bone marrow-derived macrophages (right panel) at an MOI of 10:1. (Assessed using CFU-based phagocytosis assays). (TIF) [file ppat.1007777.s004.tif]

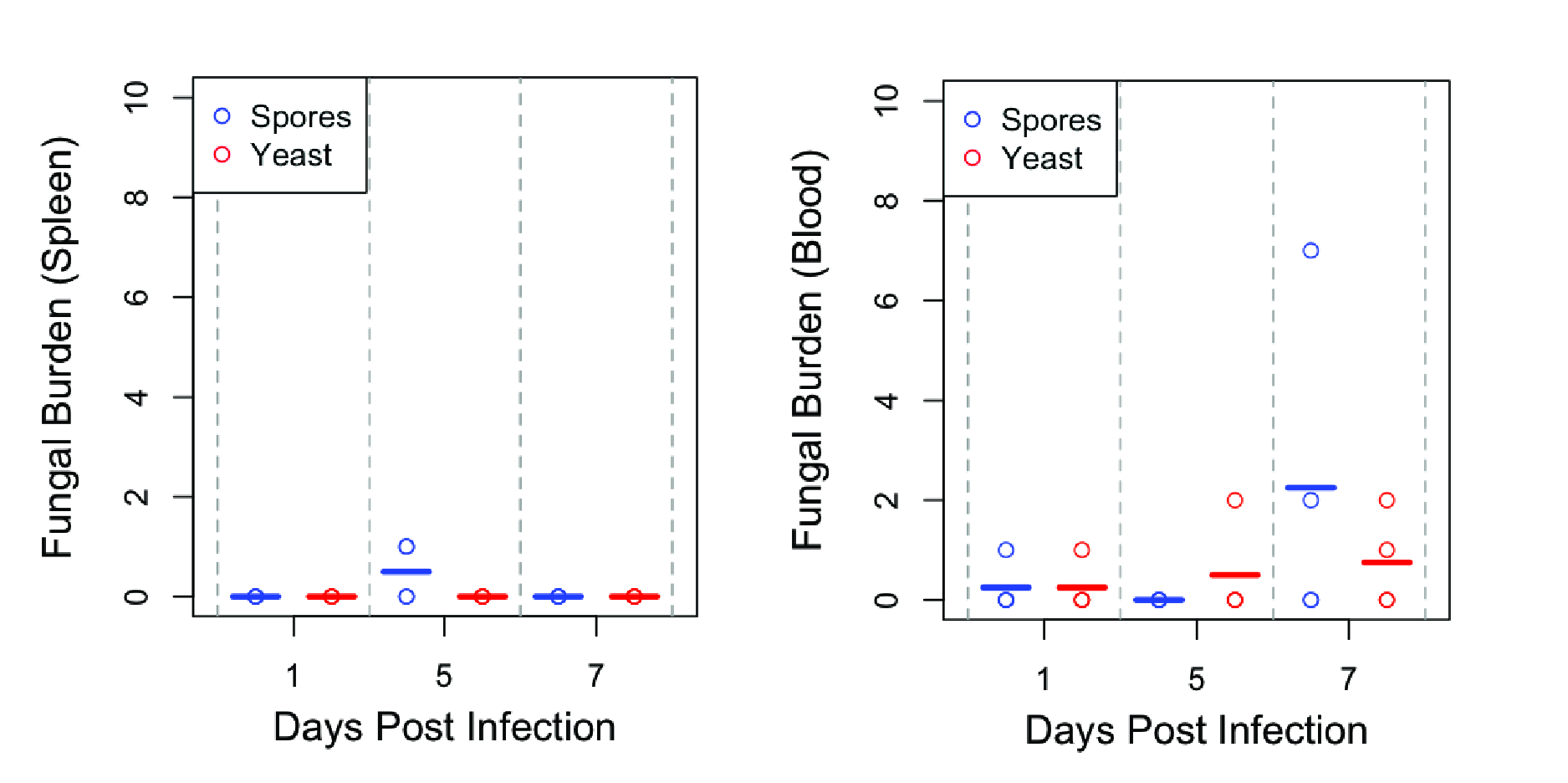

Supplement: S5 Fig — Total fungal burden (colony forming units) in the spleen and blood of mice (3 per group) infected intranasally with 1x10^6^ yeast in a 1:1 mixture of JEC20 + JEC21 (red) or 1x10^6^ spores derived from a JEC20 x JEC21 cross (blue) after 1, 5, and 7 days of infection. (TIF) [file ppat.1007777.s005.tif]

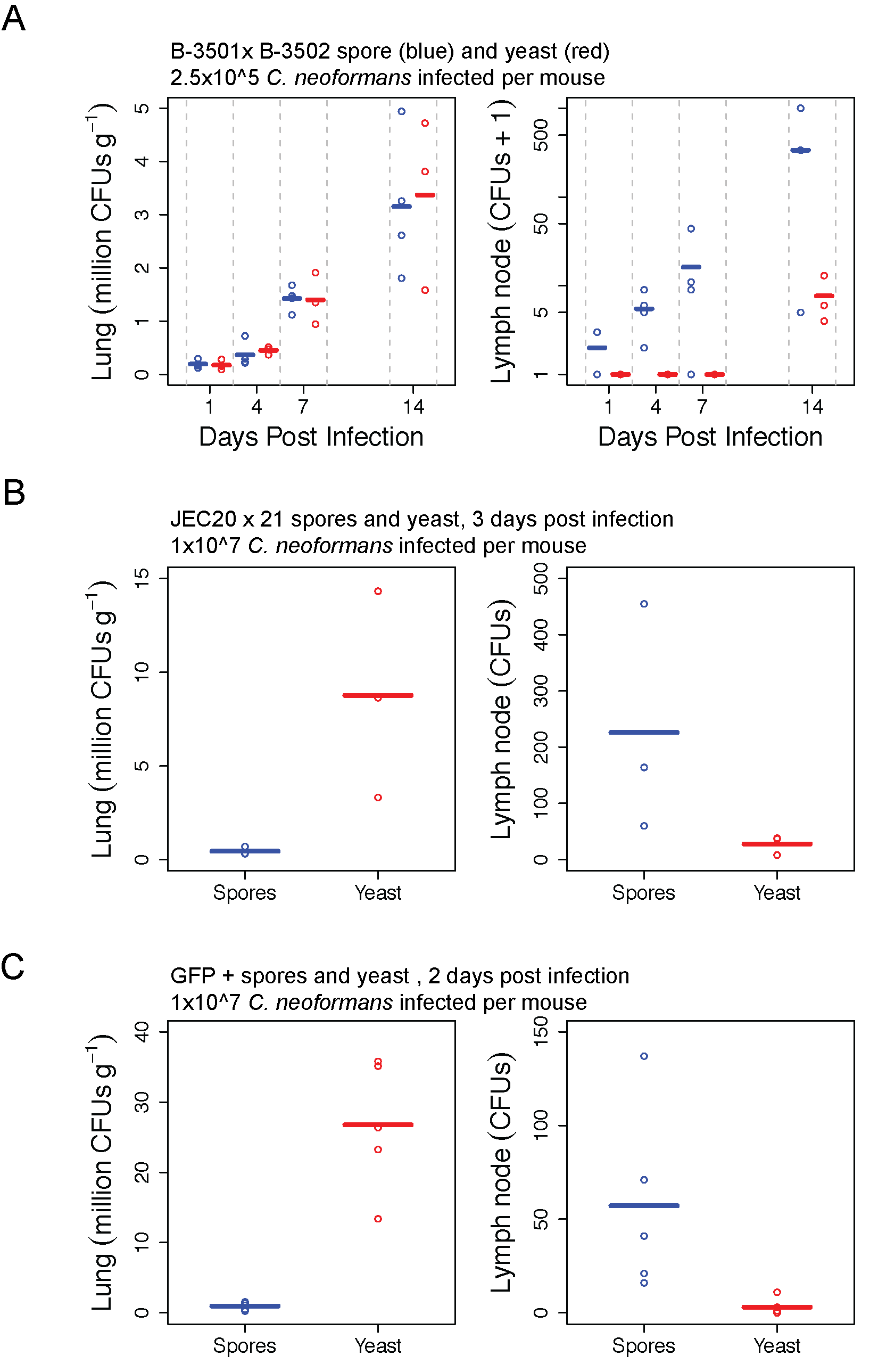

Supplement: S6 Fig — Lung and mediastinal lymph node fungal burdens early in infection for mice infected with spores (blue) or yeast (red) from various strains and inocula of Cryptococcus (as indicated above figure panels for A and B). Panel C used GFP+ derivatives of JEC20 and JEC21 (strains CHY3955 and CHY3952). (PNG) [file ppat.1007777.s006.png]

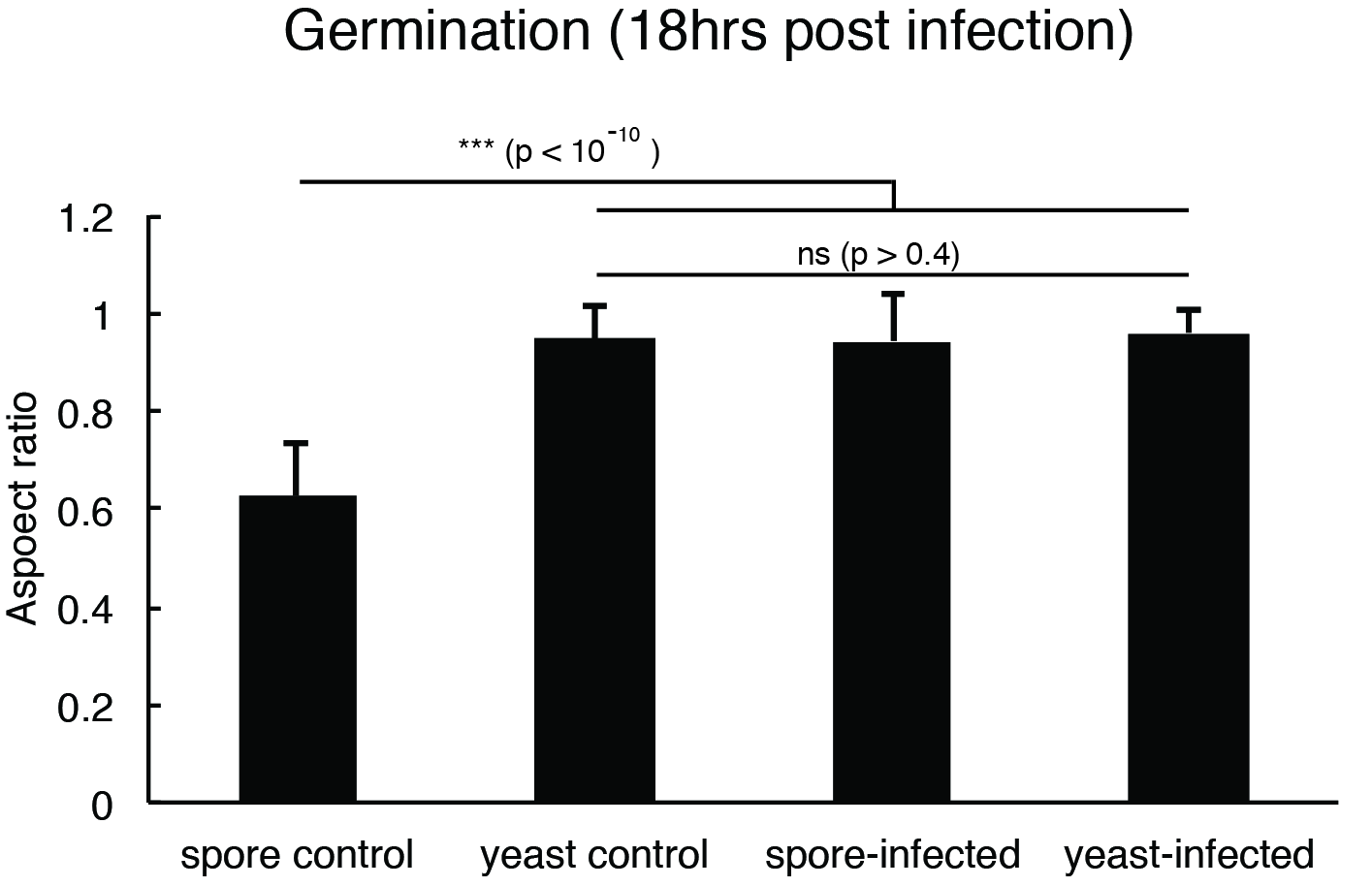

Supplement: S7 Fig — Germination state of cryptococcal cells recovered from spore- or yeast-infected mice 18 hours post-infection compared to the measured germination state of spore and yeast controls in vitro. Bars show average value of 20 cryptococcal cells measured per control or per infected mouse (n = 3 mice for spore-infected). Error bars show SEM, and p-values were calculated using a Student’s t-test. (TIF) [file ppat.1007777.s007.tif]

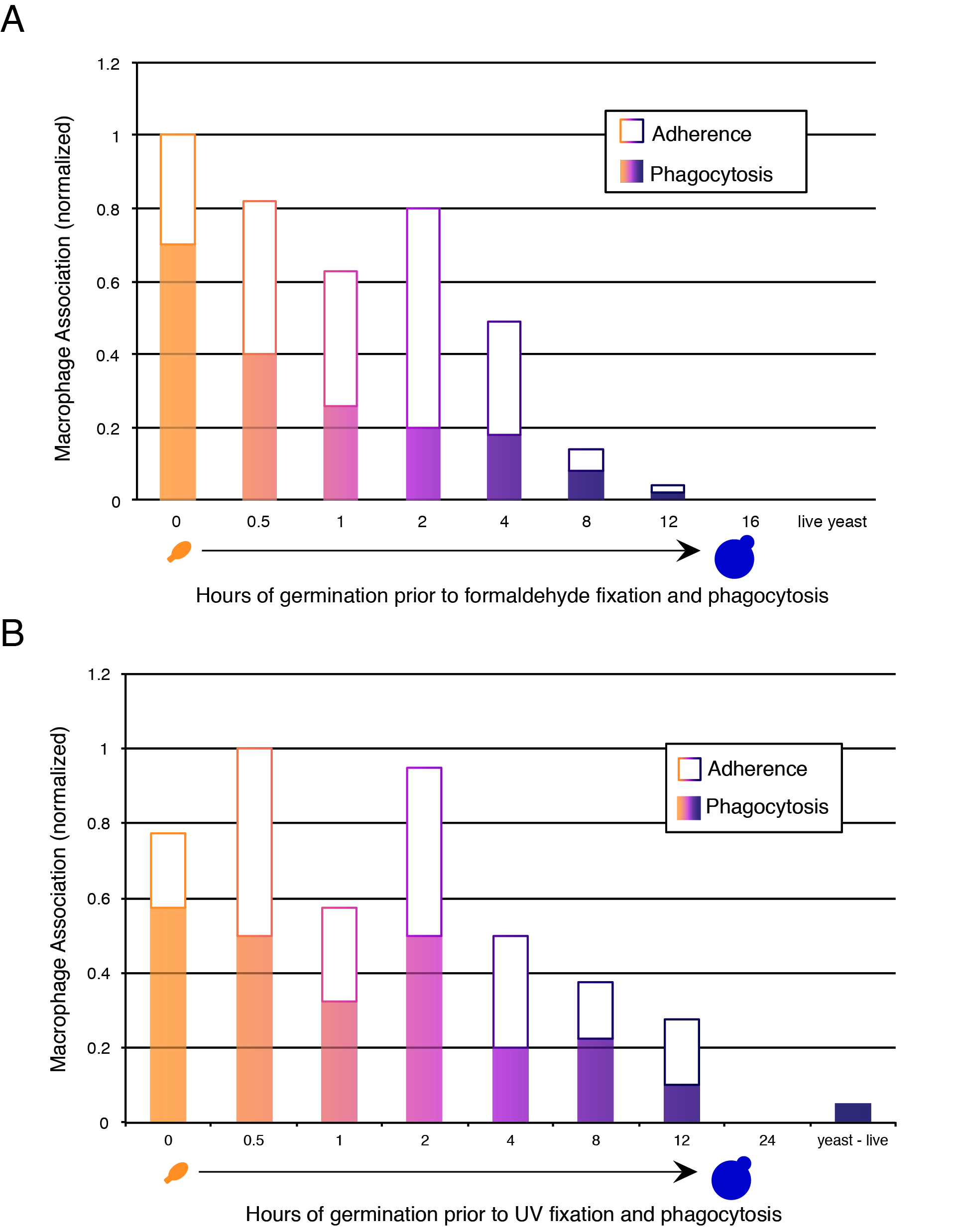

Supplement: S8 Fig — Relationship between germination state of cryptococcal cells and association (adherence and phagocytosis) with RAW 264.7 macrophages. Cryptococcal cells were fixed using (A) formaldehyde or (B) UV at various time-points during germination, and adherence and phagocytosis were assessed microscopically. (TIF) [file ppat.1007777.s008.tif]

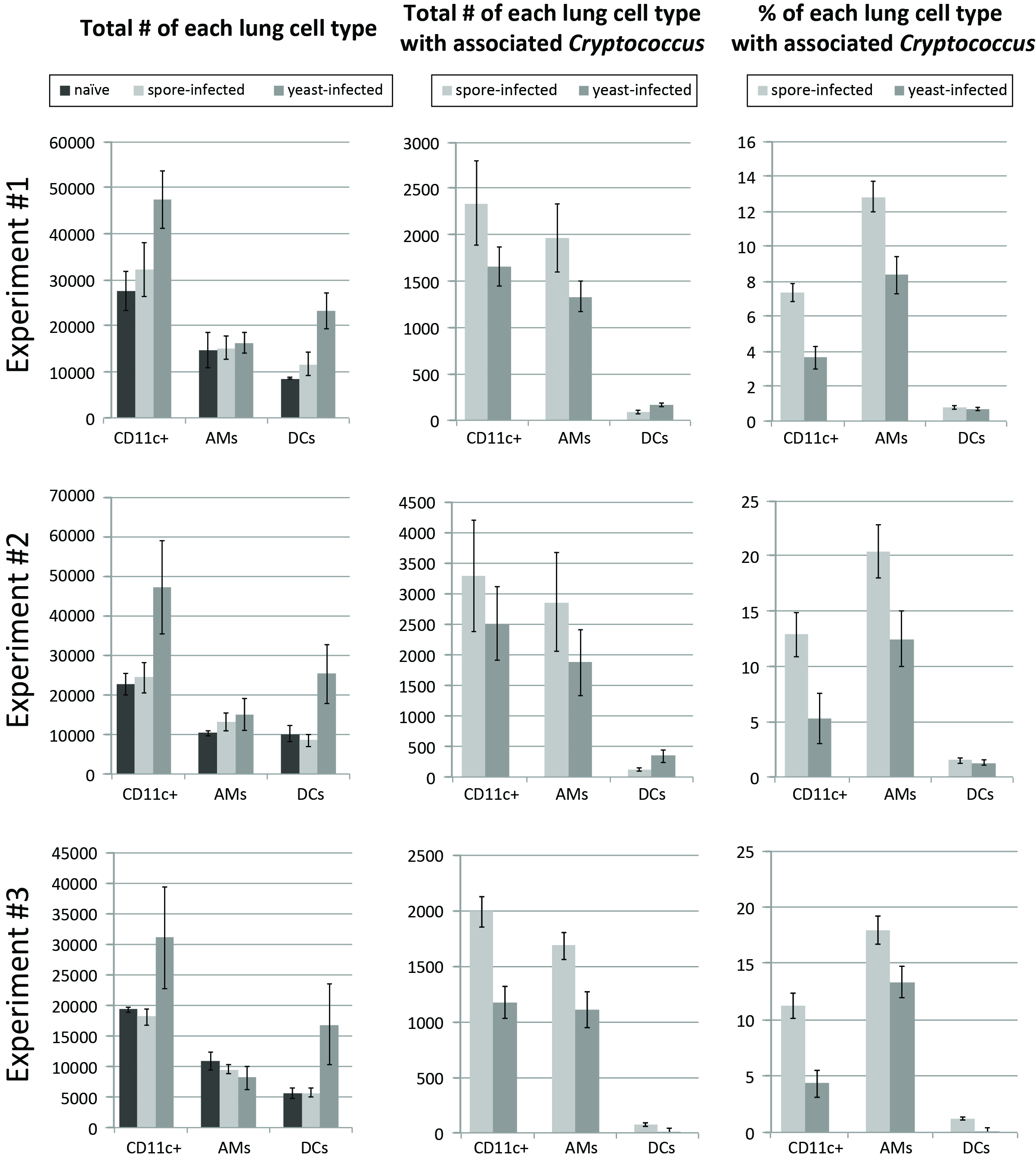

Supplement: S9 Fig — Biological replicates showing that CD11c+ cells display increased association with cryptococcal spores compared to yeast at 6 hours post-infection in the mouse lung. (TIF) [file ppat.1007777.s009.tif]
