# Supplementary material for: Infectious particle identity determines dissemination and disease outcome for the inhaled human fungal pathogen Cryptococcus
Source: PLoS Pathog. 2019 Jun 27;15(6):e1007777. doi: 10.1371/journal.ppat.1007777 (PMC6597114; doi:10.1371/journal.ppat.1007777)
Supplement: S1 Table — (DOCX) [file ppat.1007777.s010.docx]

| **Primer Name** | **Sequence (5’ to 3’)** | **Description/product** |
| --- | --- | --- |
| CHO5360 | TGA TTA CGC CAA GCT GTC GAC ACA AGA TGT TTT ACA TCA GTA TCG TCG | FWD - safe haven left flank amplification + pCH1227 homology |
| CHO5361 | GCT CGG TAC CAA GCT TGC CCA AAG ACT TCG AGT CAC | REV - safe haven left flank amplification + pCH1227 homology |
| CHO5362 | AGC ATG CAT CTA GAG GGC CCA AGC TTG GCC AAT AAA GCA CGT CCA A | FWD - safe haven right flank amplification + pCH1227 homology |
| CHO5363 | CTA TAG GGC GAA TTG GTC GAC ACC AAA CTA ATC TGT GTC AAA ATG GAT | REV - safe haven right flank amplification + pCH1227 homology |
| CHO5368 | GCG CGT TGG CCG ATT C | pCH1351 (plasmid) FWD Sequencing primer Left flank 5’ junction |
| CHO5369 | GAA GCC CTT TAG GTC TGA TCG | pCH1351 (plasmid) REV Sequencing primer Left flank 5’ junction |
| CHO5370 | CGG TCT CAG AGT GCC AC | pCH1351 (plasmid) FWD Sequencing primer Left flank 3’ junction |
| CHO5371 | CCG TAA CGC CGA TTT GAA GTG | pCH1351 (plasmid) REV Sequencing primer Left flank 3’ junction |
| CHO5372 | GTT GTT ACC ATC ATC CTC TCC TCC | pCH1351 (plasmid) FWD Sequencing primer right flank 5’ junction |
| CHO5372 | GTG GAG ATT ACT GGT ACC TGT CT | pCH1351 (plasmid) REV Sequencing primer right flank 5’ junction |
| CHO5374 | GGG GCC AAA GAT CCG AG | pCH1351 (plasmid) FWD Sequencing primer right flank 3’ junction |
| CHO5374 | CGC TTA ATG CGC CGC | pCH1351 (plasmid) REV Sequencing primer right flank 3’ junction |

***S1 Table.*** Primers used for cloning pCH1351.
