# Supplementary material for: Infectious particle identity determines dissemination and disease outcome for the inhaled human fungal pathogen Cryptococcus
Source: PLoS Pathog. 2019 Jun 27;15(6):e1007777. doi: 10.1371/journal.ppat.1007777 (PMC6597114; doi:10.1371/journal.ppat.1007777)
Supplement: S2 Table — (DOCX) [file ppat.1007777.s011.docx]

| **Primer Name** | **Sequence (5’ to 3’)** | **Description/product** |
| --- | --- | --- |
| CHO5394 | CCC GCT AAC TTT CTA CCT GGT C | Primer to assess proper integration of transgene constructs in the genomic safe harbor on chromosome 1 |
| CHO5395 | CAC CAC AAC ACA TCT ATC ACC ATG GTG AGC AAG GGC GAG GAG G | Amplify mCherry with left flank overhang |
| CHO5396 | CGG CAT CCT TCT TGG CGG ATC CCT TGT ACA GCT CGT CCA TGC CGC CGG | Amplify mCherry with right flank overhang |
| CHO5397 | CCT CCT CGC CCT TGC TCA CCA TGG TGA TAG ATG TGT TGT GGT G | Amplify Left Flank with mCherry overhang (use with CHO5360, See Table S1) |
| CHO5398 | CCG GCG GCA TGG ACG AGC TGT ACA AGG GAT CCG CCA AGA AGG ATG CCG | Amplify Right Flank with mCherry overhang (Use with CHO5363, See Table S1) |
| CHO5399 | GCT AGT TTC TAC ATC TCT TCc tag caa aag tga ctc tat tca agg gc | Amplify Right Flank with NEO cassette overhang |
| CHO5400 | GCT AGT TTC TAC ATC TCT TCc tag caa aag tga ctc tat tca agg gc | Amplify NEO cassette and mCherry with Right Flank Overhang |
| CHO5401 | CGG CAT CCT TCT TGG CGG ATC CCT TGT ACA GCT CGT CCA TGC CGC CGG | Amplify targeted mCherry cassette out of the crypto genome and make an overlap PCR product of pH3::mCherry::NLS::NAT. This primer sits at the c-terminus of mCherry with NLS overlap sequence. |
| CHO5402 | CCG GCG GCA TGG ACG AGC TGT ACA AGG GAT CCG CCA AGA AGG ATG CCG | Amplify targeted mCherry cassette out of the crypto genome and make an overlap PCR product of pH3::mCherry::NLS::NAT. This primer sits at the N-terminus of NLS with mCherry overlap sequence. |
| CHO5403 | CAC TAT AGG GCG AAT TGG GCC CAT CAT CAC GCC ACA CCC GGT AAC C | Amplify targeted mCherry cassette out of the crypto genome and make an overlap PCR product of pH3::mCherry::NLS::NAT. This primer sits at the 3' end of NLS and amplified NLS |
| CHO5404 | GGT TAC CGG GTG TGG CGT GAT GAT GGG CCC AAT TCG CCC TAT AGT G | Amplify targeted mCherry cassette out of the crypto genome and make an overlap PCR product of pH3::mCherry::NLS::NAT. This primer sits at the 3' end of NEO cassette and amplifies right flank and NEO cassette. |

***S2 Table.*** Primers used for the creation of strains CHY4028 and CHY4031.
